# Development of selective protease inhibitors *via* engineering of the bait region of human α_2_-macroglobulin

**DOI:** 10.1016/j.jbc.2021.100879

**Published:** 2021-06-15

**Authors:** Seandean Lykke Harwood, Nadia Sukusu Nielsen, Khang Diep, Kathrine Tejlgård Jensen, Peter Kresten Nielsen, Kazuhiro Yamamoto, Jan J. Enghild

**Affiliations:** 1 Department of Molecular Biology and Genetics, Aarhus University, Aarhus, Denmark; 2 Global Research Technologies, Novo Nordisk A/S, Måløv, Denmark; 3 Institute of Life Course and Medical Sciences, University of Liverpool, Liverpool, United Kingdom

**Keywords:** α_2_-macroglobulin, protease, proteinase, proteolysis, metalloprotease, protease inhibition, inhibition mechanism, mutagenesis *in vitro*, protein engineering, αM, Alpha-macroglobulin protein superfamily, α’NT, The N-terminal region of the truncated α chain, A2M, α2-macroglobulin, A2ML1, A2M-like protein 1, ADAMTS, A disintegrin and metalloproteinase with thrombospondin motifs, BAPN, 3-aminopropanenitrile, BR, Bait region, CUB, C1r/C1s, urchin embryonic growth factor, and bone morphogenetic protein 1, DQ, 1,4-diethyl-decahydro-quinoxaline, ETD, Electron transfer dissociation, HBS, HEPES-buffered saline, here defined as 20 mm HEPES-NaOH, 150 mM NaCl, pH 7.4, HCD, High-energy collision-induced dissociation, LNK, Linker region, LRP1, Low-density lipoprotein receptor-related protein 1, MA, Methylamine, MG, Macroglobulin domain, MMP, Matrix metalloprotease, PMSF, Phenylmethanesulfonyl fluoride, PZP, Pregnancy zone protein, SDS-PAGE, sodium dodecyl sulfate-polyacrylamide gel electrophoresis, TE, Thiol ester domain, TR, The *tabula rasa* bait region

## Abstract

Human α_2_-macroglobulin (A2M) is an abundant protease inhibitor in plasma, which regulates many proteolytic processes and is involved in innate immunity. A2M’s unique protease-trapping mechanism of inhibition is initiated when a protease cleaves within the exposed and highly susceptible “bait region.” As the wild-type bait region is permissive to cleavage by most human proteases, A2M is accordingly a broad-spectrum protease inhibitor. In this study, we extensively modified the bait region in order to identify any potential functionally important elements in the bait region sequence and to engineer A2M proteins with restrictive bait regions, which more selectively inhibit a target protease. A2M in which the bait region was entirely replaced by glycine-serine repeats remained fully functional and was not cleaved by any tested protease. Therefore, this bait region was designated as the “*tabula rasa*” bait region and used as the starting point for further bait region engineering. Cleavage of the *tabula rasa* bait region by specific proteases was conveyed by the insertion of appropriate substrate sequences, *e.g.*, basic residues for trypsin. Screening and optimization of *tabula rasa* bait regions incorporating matrix metalloprotease 2 (MMP2) substrate sequences produced an A2M that was specifically cleaved by MMPs and inhibited MMP2 cleavage activity as efficiently as wild-type A2M. We propose that this approach can be used to develop A2M-based protease inhibitors, which selectively inhibit target proteases, which might be applied toward the clinical inhibition of dysregulated proteolysis as occurs in arthritis and many types of cancer.

Proteases constitute the largest enzyme family in humans with 641 currently identified members (1). In addition, they are common virulence factors produced by viruses, bacteria, and parasites. As they are involved in virtually every type of biological event and pathogenic in many diseases, they represent potential drug targets (1). However, the therapeutic targeting of proteases, such as matrix metalloproteases (MMPs) in cancer, has proven difficult because of broad protease inhibition by small-molecule therapeutics (2, 3, 4). The development of new therapeutics with restricted inhibitory activities that selectively target individual proteases has the potential to both improve the efficacy and alleviate toxicities in protease-targeting treatment strategies.

Human α_2_-macroglobulin (A2M) is a 720 kDa homotetrameric protein that is abundant in plasma and is archetypical for the alpha-macroglobulin (αM) protease inhibitor family, whose human members also include A2M-like protein 1 (A2ML1) and pregnancy zone protein (PZP). A2M has a unique mechanism of inhibition where it collapses around proteases and irreversibly sequesters them from protein substrates (5). This conformational collapse is triggered by peptide bond hydrolysis within the bait region, a 39-residue sequence that is unstructured and poorly evolutionarily conserved, but is rapidly and preferentially cleaved by most proteases regardless of their catalytic class (6, 7). Protease trapping is associated with covalent conjugation of the protease by a thiol ester bond that is initially buried within A2M and exposed upon its proteolysis-induced conformational change; although covalent conjugation is required for protease trapping by the monomeric αMs, tetramers such as A2M can trap proteases noncovalently (8, 9, 10, 11). As a consequence of its conformational change, A2M reveals a binding site that interacts with a scavenger receptor, low-density lipoprotein receptor-related protein 1 (LRP1), and this interaction quickly removes A2M-protease complexes from circulation through internalization and endosomal degradation, primarily in the liver (12, 13).

In order for a protease to be inhibited by an αM protease inhibitor, the protease must be able to cleave within the αM’s bait region. The bait region of A2M contains 15 of the 20 canonical amino acids, permitting cleavage by most proteases with simple substrate recognition (*e.g.*, only involving the P1 or P1’ residue) and also has an MMP cleavage site (14, 15) (sequence: PEGL) (Fig. 1*A*). *In vitro*, A2M has been shown to inhibit proteases involved in coagulation and fibrinolysis, *e.g.*, plasma kallikrein, thrombin, FXa, and plasmin (7, 16, 17, 18), inflammatory proteases, *e.g.*, cathepsin G, human neutrophil elastase, and mast cell chymase (19, 20), and proteases that remodel the extracellular matrix, *e.g.*, MMPs and ADAMTS (a disintegrin and metalloproteinase with thrombospondin motifs) proteases (14, 15, 21). A2M can also inhibit pathogen-expressed proteases, including *Porphyromonas gingivalis* gingipain R, *Staphylococcus aureus* GluC (a.k.a. V8), and HIV protease 1, and delivers these potential antigens to LRP1-expressing antigen-presenting cells such as macrophages and dendritic cells (22, 23, 24, 25). The addition of protease substrate sequences for proteases that are not inhibited by wild-type A2M into the bait region is sufficient to allow their inhibition, as has been demonstrated for furin, tobacco etch virus protease, and LysC (26, 27, 28).

**Figure 1:**
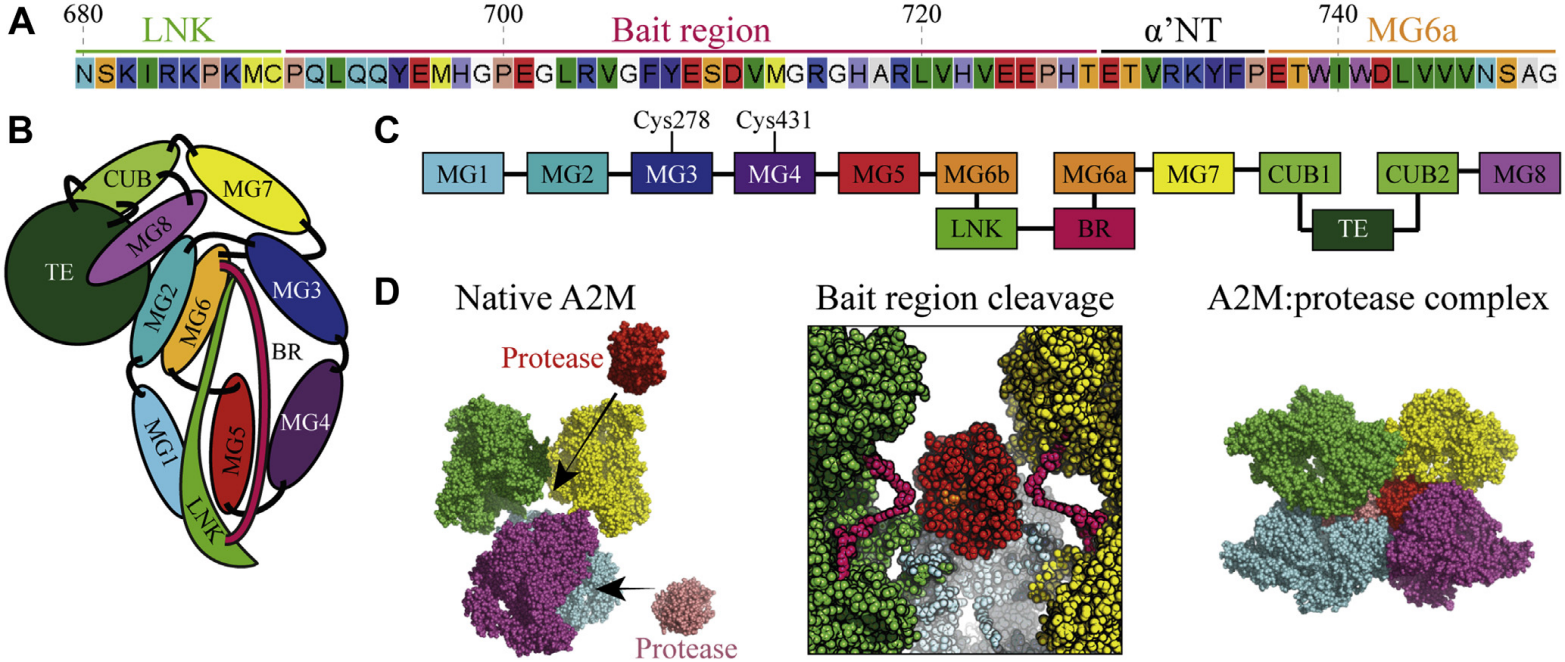
**A2M’s bait region sequence, domain organization, and mechanism of action.***A*, the bait region sequence of A2M (residues 690–728), as well as the beginning and end of the adjacent MG6a and LNK domains. *B*, a schematic representation of the domain configuration in a native A2M subunit. *C*, the domains of an A2M subunit. *D*, the inhibitory mechanism of A2M, shown using models derived from negative stain electron microscopy, small-angle X-ray scattering, and cross-linking mass spectrometry (41). A2M consists of four identical subunits (colored separately). Proteases must enter native A2M in order to cleave its bait region, which triggers a collapse of A2M that engulfs the protease. Up to two proteases can be trapped in close succession.

The αM protein superfamily includes the complement factors C3, C4, and C5, which are homologous to A2M and also undergo proteolysis-induced conformational changes (29). αM complement factors and the four subunits of the A2M homotetramer have a conserved structure, which includes eight macroglobulin (MG) domains, a CUB domain, and the thiol ester (TE) domain (Fig. 1, *B* and *C*) (30). Crystal structures of each complement factor in its native and protease-cleaved conformation have been determined (31, 32, 33, 34, 35, 36, 37), but not for A2M. A2M can be made to collapse into a conformation that is highly similar to its protease-cleaved conformation by aminolysis of its thiol ester (*e.g.*, using methylamine (MA)) (5, 38, 39), and the crystal structure of A2M-MA has been determined as a surrogate for its protease-cleaved conformation (40). We have recently developed a low-resolution model of native A2M using negative stain electron microscopy, small-angle X-ray scattering, and cross-linking mass spectrometry (41). This model indicates that proteases must enter the interior of native A2M to access and cleave the bait regions, at which point A2M collapses and closes off its entrances, thereby trapping the intruding protease (Fig. 1*D*). The bait regions occupy the interior space of native A2M and may interact with each other, as suggested by the formation of intersubunit disulfides upon the introduction of cysteine residues into the bait region (42).

The precise molecular mechanism by which bait region cleavage triggers A2M’s conformational collapse is not known. In the αM complement factors, the anaphylactic domain corresponds to the bait region of αM protease inhibitors, and its removal by proteolysis is thought to perturb the adjacent MG3 and MG8 domains, thereby initiating a conformational change (36). However, while the anaphylactic domain has a rigid α-helix structure (32) and is evolutionarily conserved across species, the bait region is unstructured and poorly conserved (Fig. S1), and it is unclear how it could participate in an equivalent triggering mechanism. While the reaction of A2M’s thiol ester with nucleophiles (*e.g.*, lysine side chain ε-amino groups on the surface of proteases) takes place during or after its conformational change (43), its amino- or hydrolysis is not required for the conformational change to take place, as A2M lacking a thiol ester is still induced to change its conformation by proteolysis (44), as are the thiol-ester-lacking αM proteins C5 and chicken ovostatin (10, 37).

In this study, we have produced and characterized recombinant A2M proteins with modified bait regions, which investigate the bait region’s functional role and demonstrate the design of new protease inhibitors. We found that A2M’s bait region can be replaced in its entirety with 13 Gly-Gly-Ser triplets without preventing A2M from assembling into its usual homotetrameric structure or assuming its native conformation. This *tabula rasa* bait region was not initially cleaved by any of 12 tested proteases, but could be cleaved by trypsin, LysC, or MMP2 upon introducing an appropriate cleavage site into its sequence. Bait region cleavage of *tabula rasa* A2Ms resulted in protease conjugation through A2M’s thiol ester, the induction of A2M’s conformational collapse, and protease inhibition, as demonstrated for trypsin and MMP2. An MMP2 substrate bait region that was selectively cleaved by MMPs was identified by screening using ten human proteases. Further optimization of the bait region yielded a *tabula rasa* A2M, which inhibited MMP2 as efficiently as wild-type A2M. Altogether, these results demonstrate an approach to developing more specific A2M-based protease inhibitors through bait region engineering and have identified several factors that are important during bait region design, including the cleavage site position and bait region length.

## Results

### Bait region substitution with 13 Gly-Gly-Ser triplets produces A2M that is tetrameric, native, and inducible

**Figure 2:**
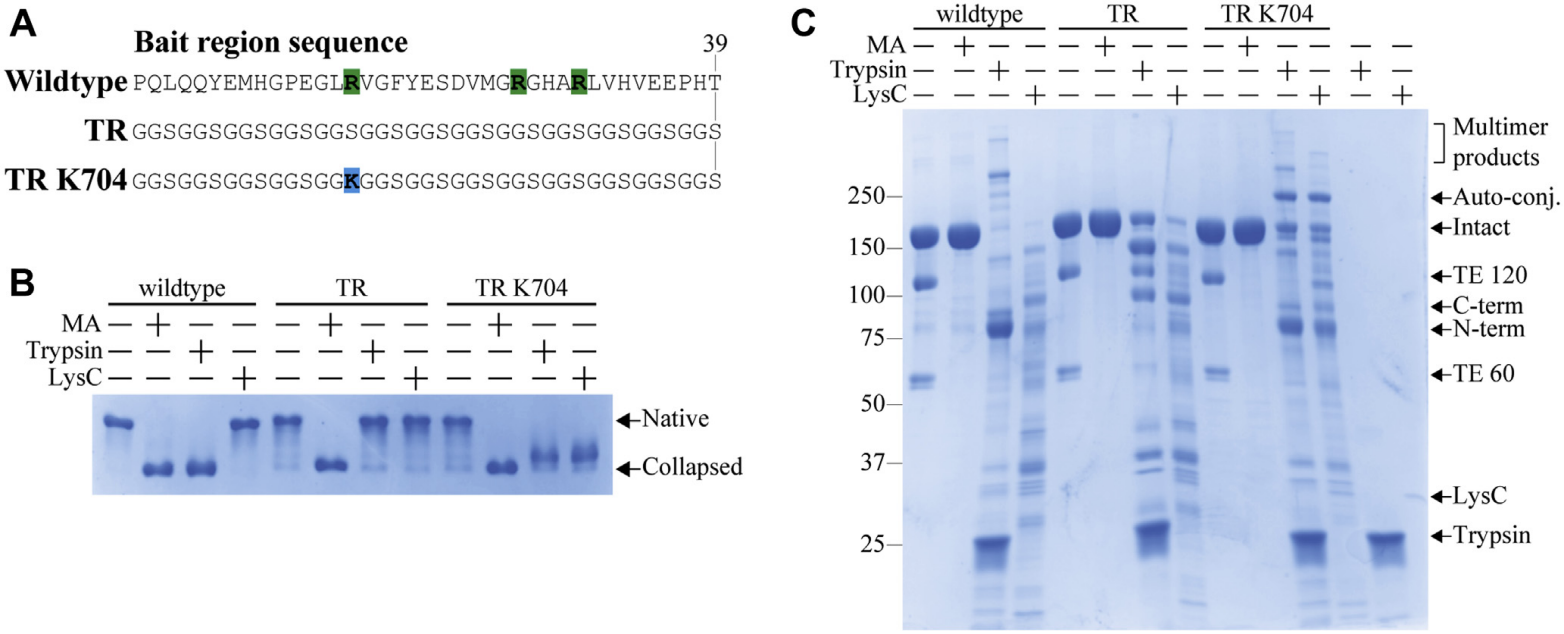
**The conformation and functionality of *tabula rasa* A2M.***A*, sequences of the wild-type, *tabula rasa* (TR), and TR K704 bait regions. Cleavage sites for trypsin (arginine and lysine residues) are indicated. *B*, pore-limited native PAGE of A2M incorporating the three given bait region sequences. As indicated, A2M samples were treated with methylamine, trypsin, or LysC. All constructs originally demonstrated the slow electrophoretic mobility that is characteristic of A2M’s native conformation; upon methylamine aminolysis or bait region cleavage, A2M collapses and demonstrates a faster electrophoretic mobility. Wild-type A2M and A2M TR K704 were both collapsed by trypsin, while only A2M TR K704 was collapsed by LysC; A2M TR was not collapsed by either protease. *C*, reducing SDS-PAGE of the same A2M samples as in panel *B*. The thiol-ester-dependent heat-fragmentation bands (TE 120 and TE 60) disappeared upon methylamine treatment. Bait region cleavage of A2M gives its ~85 and ~95 N- and C-terminal fragment bands; the C-terminal fragment additionally forms high-MW multimer products through thiol-ester-mediated conjugation. When the bait region is impermissible to trypsin or LysC cleavage, A2M is cleaved outside of the bait region without any activation if its thiol ester. A2M TR K704 forms an intense ~250 kDa band upon proteolytic activation that was determined to arise from autoconjugation, see Figure 3. SDS-PAGE, sodium dodecyl sulfate-polyacrylamide gel electrophoresis.

The bait region is both ground zero for A2M’s proteolytically induced conformational change and the major determiner of which proteases are inhibited by A2M. However, the low conservation of the bait region sequence across species (Fig. S1) suggests that it has no essential structure or sequence motifs. In order to remove essentially all protease cleavage sites or putative structural features from the bait region and determine the extent to which it tolerates modification, we entirely replaced the 39-residue bait region sequence with 13 Gly-Gly-Ser repeats, chosen for their solubility and low susceptibility to proteolysis (Fig. 2*A*).

The resulting *tabula rasa* bait region could be incorporated into recombinant A2M, resulting in a *tabula rasa* A2M protein that was predominantly tetrameric and in its native conformation, with an intact thiol ester apparent from the formation of characteristic heat-induced autolysis products in sodium dodecyl sulfate-polyacrylamide gel electrophoresis (SDS-PAGE) (Fig. 2, *B* and *C*). Upon aminolysis of its thiol ester with methylamine, *tabula rasa* A2M underwent a conformational collapse indistinguishable from that of wild-type A2M, as determined by pore-limited native PAGE; however, *tabula rasa* A2M was not cleaved in its bait region by either trypsin or LysC and remained in its native conformation despite proteolysis by these proteases outside of its bait region (Fig. 2, *B* and *C*). While the majority of *tabula rasa* A2M assembled into the normal homotetramer, dimeric *tabula rasa* A2M that had failed to tetramerize was slightly more abundant than wild-type A2M; dimers were removed during purification prior to all characterization (Fig. S2). Similarly, while the majority of *tabula rasa* A2M was in a native conformation, both fully collapsed A2M and conformations with an intermediate electrophoretic mobility were apparent in pore-limited native PAGE and more abundant than in wild-type A2M (Fig. 2*B*). Before using these proteins in quantitative protease inhibition assays, nonnative A2M was depleted using LRP1-conjugated resin (Fig. S3).

The TR K704 bait region added a lysine residue at position 704 and was cleaved by trypsin and LysC, which caused A2M to undergo a conformational collapse (Fig. 2, *A*–*C*); as the wild-type bait region and the initial *tabula rasa* bait region contain no lysine residues, LysC cleaved these A2Ms in locations other than the bait region without inducing a conformational change, as has been previously reported (45). The protease-induced conformational change of A2M TR K704 activated its thiol ester and resulted in the formation of covalent conjugation products that were apparent as high-MW bands in reducing SDS-PAGE (Fig. 2*C*). Altogether, these results showed that while the wild-type bait region may make minor contributions toward A2M’s tetrameric assembly and the assumption of its native conformation, no part of its sequence is structurally or functionally essential. The majority of *tabula rasa* A2M is tetrameric and in a native conformation and can undergo a methylamine- or proteolysis-induced conformational change (if protease substrate sites are incorporated into the bait region).

### Bait region lysine residues can be conjugated by the thiol ester upon its proteolytic activation

**Figure 3:**
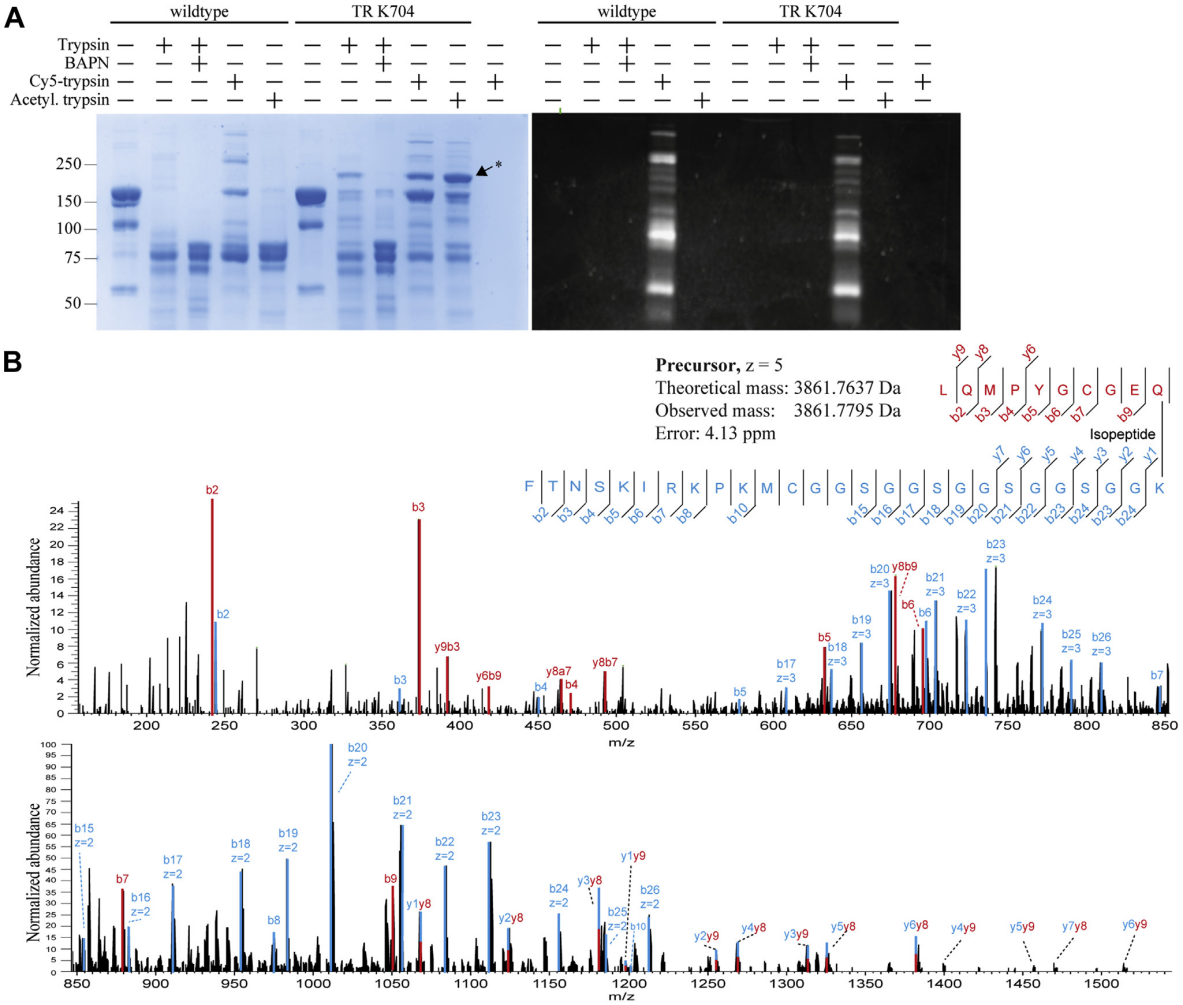
**Investigating autoconjugation to a bait region lysine residue.***A*, wild-type A2M and A2M TR K704 were digested with trypsin with and without BAPN, a 70 Da nucleophile, which competes for thiol ester conjugation, as well as with a Cy5-labeled trypsin (approximately 1:1 mol/mol dye:trypsin) and acetylated trypsin. The samples were analyzed by reducing SDS-PAGE. The investigated band (marked by an *asterisk*) was dependent on thiol-ester-mediated conjugation, as it disappeared when BAPN was present. It did not contain Cy5-labeled trypsin. It became more intense if conjugation to trypsin itself was prevented by acetylation of trypsin’s lysine residues. *B*, the suspected autoconjugation band was digested in-gel with pepsin and the peptides were analyzed by LC-MS/MS. A cross-linked peptide containing the thiol ester peptide LQMPYGCGEQN and the bait region lysine peptide FTNSKIRKPKMCGGSGGSGGSGGSGGK cross-linked together by an isopeptide bond was identified from this MS2 spectrum. *b*- and *y*-type product ions are colored according to the fragmented peptide; some product ions resulted from fragmentation in both peptides. Note that the y axis is truncated to 24% in the first panel due to the high intensity of the *b*2 ion with the sequence LQ. SDS-PAGE, sodium dodecyl sulfate-polyacrylamide gel electrophoresis.

Bait region cleavage of A2M TR K704 by both trypsin and LysC produced an intense band migrating as ∼250 kDa in reducing SDS-PAGE that was not produced by trypsin-cleaved wild-type A2M (Fig. 2*C*). The bait region is spatially close to the TE domain when A2M is proteolytically activated (Fig. 1, *B*–*D*), and we hypothesized that the nucleophilic ε-amine group of Lys704’s side chain might attack the thiol ester following proteolysis, conjugating the N- and C-terminal bait region cleavage fragments together into an aberrantly migrating ∼180 kDa product. This proposed product of Lys704 autoconjugation disappeared when 3-aminopropanenitrile (BAPN), a small nucleophile that reacts with the thiol ester after it is proteolytically exposed and out-competes conjugation to other nucleophiles (9), was included alongside trypsin, demonstrating that it is formed through thiol-ester-mediated conjugation (Fig. 3*A*). After cleavage of A2M TR K704 with Cy5-labeled trypsin, this band was not fluorescent and therefore does not contain trypsin (Fig. 3*A*). In fact, the product band became more intense upon cleaving A2M TR K704 with acetylated trypsin, which is not efficiently conjugated as it lacks primary amine groups (Fig. 3*A*). LC-MS/MS analysis of the pepsin-digested product band identified a cross-linked peptide containing an isopeptide cross-link between the thiol ester Gln975 and the bait region Lys704 (Fig. 3*B*). Furthermore, the band was not produced upon tryptic cleavage of *tabula rasa* A2M with a bait region arginine residue instead of a lysine residue (Fig. S4, *A* and *B*). These results conclusively show that this band represents an autoconjugation product of the thiol ester and a bait region lysine residue.

### Tabula rasa A2M proteins inhibit trypsin in a manner that is dependent on covalent conjugation

**Figure 4:**
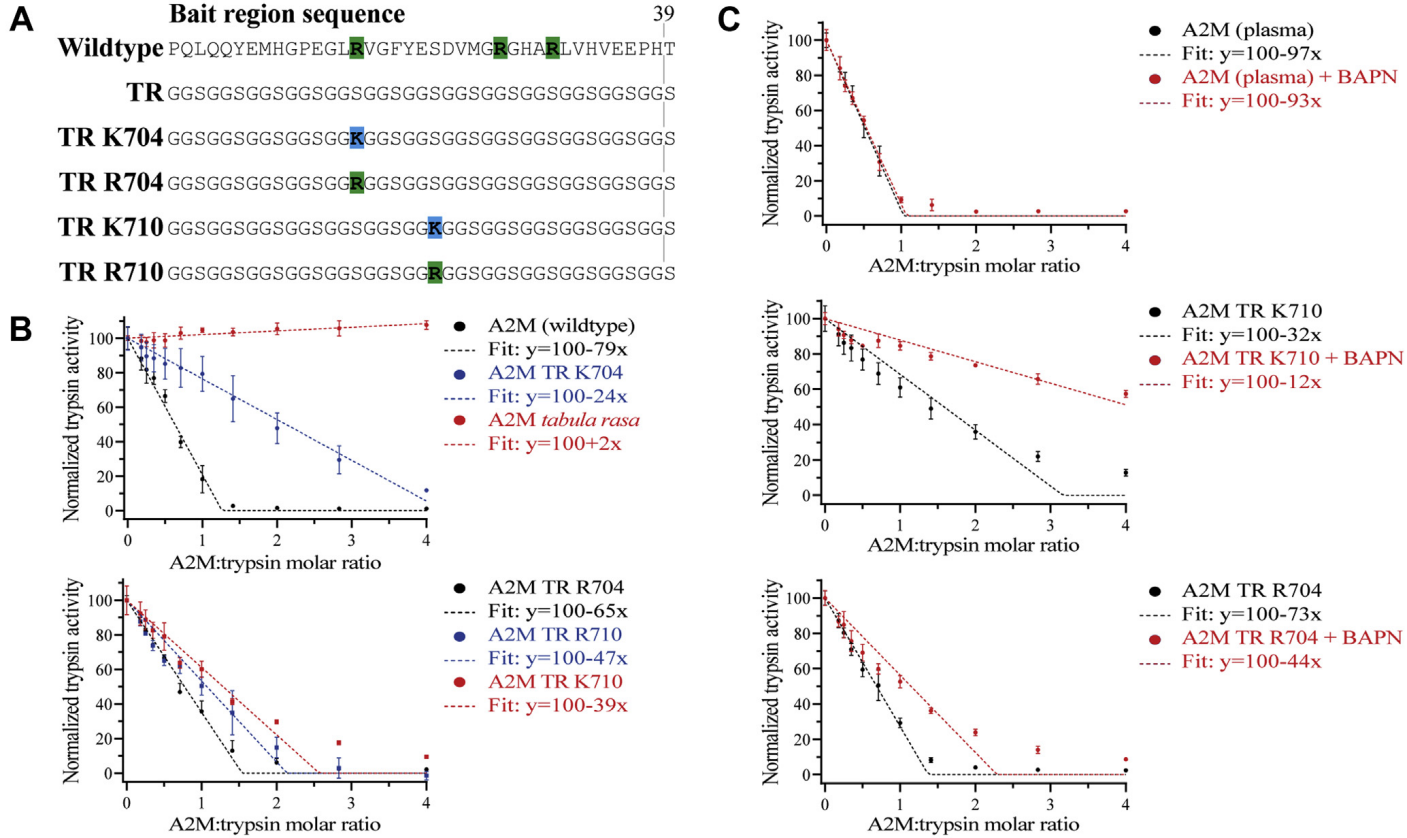
**Inhibition of trypsin by *tabula rasa*–based A2Ms.***A*, bait region sequences for all A2M proteins for which the inhibition of trypsin was determined. *B*, the inhibition of trypsin’s cleavage activity toward DQ-labeled gelatin by the indicated A2M mutants was determined. Fitting of the experimental data points for each mutant was performed by linear regression. Error bars show the standard deviation and each sample was prepared in triplicate. *C*, the inhibition of trypsin by plasma-purified A2M, A2M TR K710, and A2M TR R704 was assessed with and without BAPN, which saturates A2M’s thiol ester and out-competes the covalent conjugation of other nucleophiles. Except for the use of BAPN where noted, the experiment and analysis were performed as in panel (*B*).

We proceeded to investigate the trypsin-inhibiting ability of *tabula rasa*-based A2Ms using a fluorescently labeled gelatin substrate. In addition to A2M TR K704, bait regions incorporating an Arg704, Lys710, or Arg710 residue were produced (Fig. 4*A*). These A2M proteins appeared similar to A2M TR K704 during their initial characterization by electrophoresis (Fig. S4, *A* and *B*). In order to quantitatively determine the inhibitory capacity of the native *tabula rasa* A2Ms, nonnative A2M was depleted from these proteins using LRP1-conjugated resin before all inhibitory assays (Fig. S3). A2M with the *tabula rasa* bait region was completely unable to inhibit trypsin, but incorporation of either lysine or arginine into the bait region conveyed inhibition of trypsin (Fig. 4*B*). However, all four *tabula rasa* A2M with basic residues incorporated into their bait regions showed a decreased inhibitory capacity relative to recombinant wild-type A2M or plasma-purified A2M. *Tabula rasa* A2M with an arginine residue at position 704 or 710 possessed approximately 80% and 60% of the wild-type inhibitory capacity, respectively, while a lysine residue at these positions conveyed approximately 30% and 50% inhibition (Fig. 4*B*).

To investigate the mechanistic explanation for these decreased inhibitory capacities, trypsin inhibition assays were repeated for plasma-purified A2M, A2M TR K710, and A2M TR R704 with and without BAPN, which out-competes covalent conjugation of other nucleophiles by A2M’s proteolytically activated thiol ester (9). The inhibition of trypsin by plasma-purified A2M was not affected by BAPN, showing that its inhibition is independent of covalent conjugation as has been previously reported (9), whereas both A2M TR K710 and A2M TR R704 demonstrated much lower inhibitory capacities in the presence of BAPN (Fig. 4*C*). These results show that trypsin inhibition by *tabula rasa* A2Ms is dependent on covalent protease conjugation, in contrast to wild-type A2M.

### Identification of an MMP2-cleavable bait region sequence with improved selectivity

**Figure 5:**
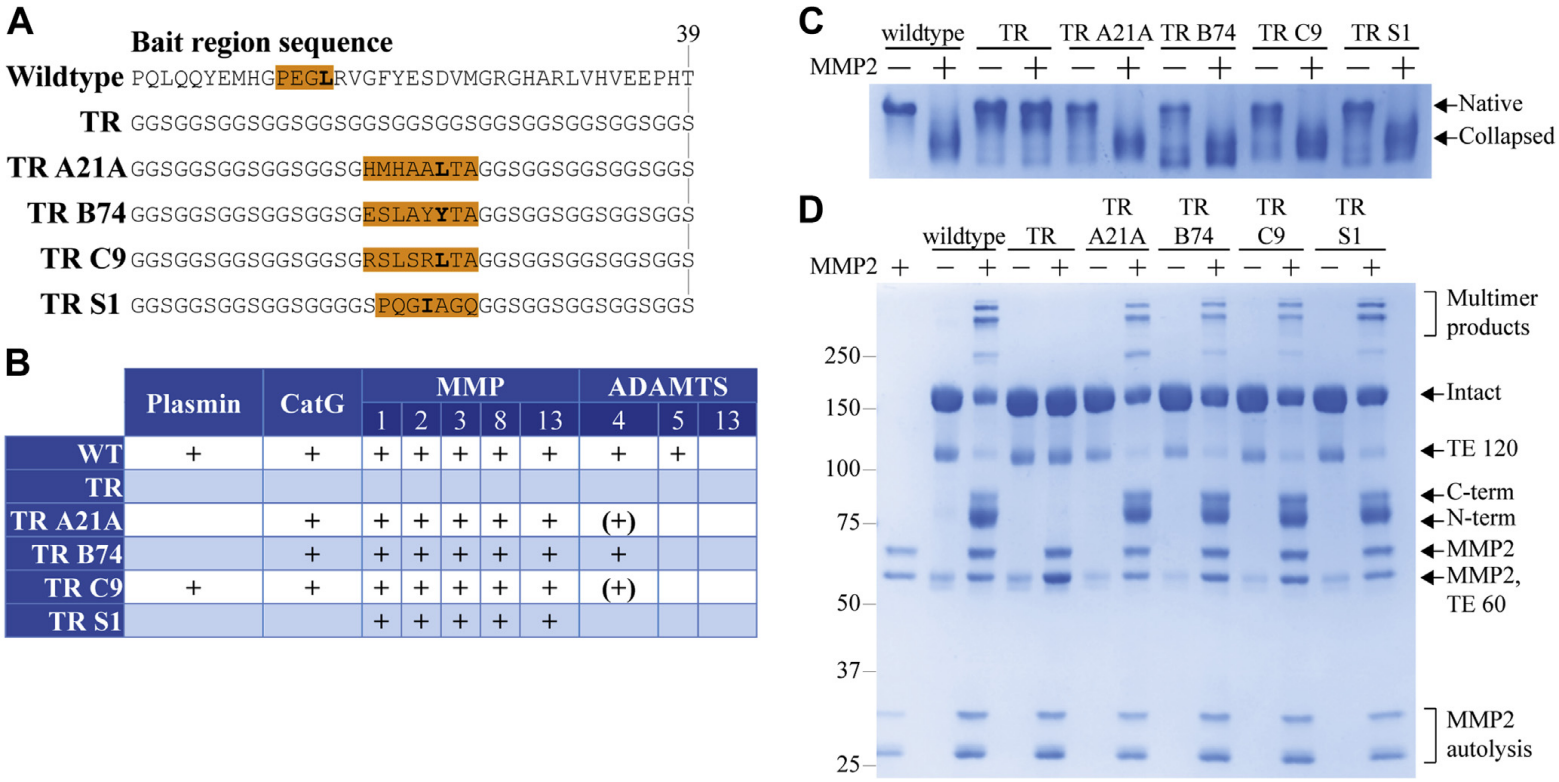
**Incorporation of MMP2 substrate sites into *tabula rasa* A2M.***A*, bait region sequences for wild-type A2M, *tabula rasa* A2M, and four TR bait regions each incorporating a different MMP2 substrate sequence (A21A, B74, C9, and S1). The MMP2 recognition sequence is colored orange; cleavage occurs at the N-terminus of the hydrophobic residue that is *bolded*. *B*, A2Ms with these six bait regions were digested by MMP2 and nine other human proteases (see Fig. S6). Proteases that are able to cleave a bait region are indicated with a + in the case of full cleavage and (+) in the case of partial cleavage. The *tabula rasa* bait region was not cleaved by any tested protease, whereas each MMP2 substrate was cleaved by every tested MMP. The TR S1 bait region was not cleaved by proteases other than MMPs, indicating an increased selectivity of inhibition relative to the wild-type bait region. *C* and *D*, pore-limited native PAGE and reducing SDS-PAGE, respectively, of the six A2Ms with and without MMP2 cleavage. All constructs are similarly bait region cleaved by MMP2, resulting in a conformational collapse and the appearance of high-MW multimer products in SDS-PAGE, with the exception of A2M TR. SDS-PAGE, sodium dodecyl sulfate-polyacrylamide gel electrophoresis.

After verifying that *tabula rasa* A2Ms retained the ability to inhibit proteases using the model protease trypsin, we designed four bait regions incorporating substrate sequences for human MMP2 (Fig. 5*A*), a potential therapeutic target in several types of cancer including ovarian cancer and pancreatic adenocarcinoma (46). Three of these substrate sequences (A21A, B74, and C9) were previously identified as MMP2 substrates using phage display (47), while the fourth (S1) is a generic MMP substrate. All four *tabula rasa*–based MMP2 substrate bait regions and the wild-type bait region were cleaved by MMP2, but not the initial A2M *tabula rasa* (Fig. 5, *B*–*D*). Despite the rapid formation of A2M-MMP2 complexes (Fig. S5*A*), A2M was never fully bait region cleaved even by a large excess of MMP2 (Fig. S5*B*), and A2M:MMP2 complexes migrated with an intermediate electrophoretic mobility (Fig. S5*C*). These results resemble A2M’s reaction with plasmin (48, 49, 50) and may be a common feature of A2M:protease complex formation with larger proteases (>60 kDa). The incomplete bait region cleavage and intermediate electrophoretic mobility of A2M:MMP2 complexes were the same for wild-type A2M and the four *tabula rasa* A2Ms with MMP2 substrate sequences.

We then tested whether the four MMP2 substrate bait regions were cleaved by nine additional human proteases (plasmin, cathepsin G, MMP1, MMP3, MMP8, MMP13, ADAMTS4, ADAMTS5, and ADAMTS13) using reducing SDS-PAGE to assess bait region cleavage and the formation of high-MW conjugation products (Fig. S6). All assessed proteases except ADAMTS13 (which is highly specific toward von Willebrand factor (51, 52)) were able to cleave wild-type A2M, while none were able to cleave A2M *tabula rasa* (Fig. 5*B*). The incorporation of any of the four MMP2 substrate sequences into the *tabula rasa* bait region conveyed cleavage by all tested MMPs, but they were differently cleaved by non-MMP proteases; for example, the C9 substrate was the only one containing an arginine residue and was the only A2M to be cleaved by plasmin (Fig. 5, *A* and *B*). Notably, the S1 substrate was only cleaved by MMPs (Fig. 5, *A* and *B*), and the A2M TR S1 protein was therefore selected for further optimization as an MMP inhibitor with improved specificity relative to wild-type A2M.

### The native content of tabula rasa–based A2Ms is improved by shortening the bait region by seven residues or restoring the ten C-terminal wild-type residues

**Figure 6:**
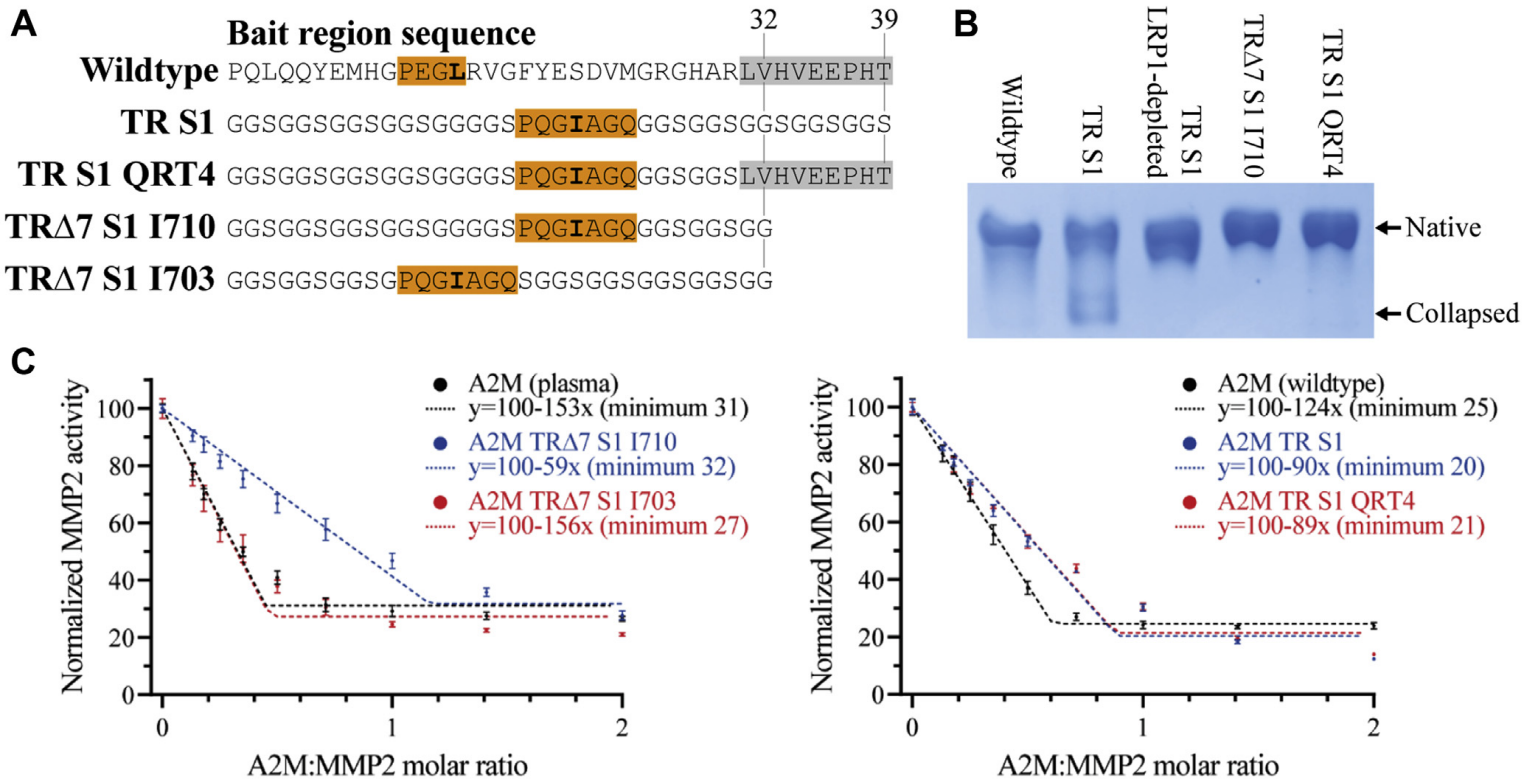
**Optimization of the production and inhibitory capacity of A2M TR S1.***A*, several modifications of the MMP2 substrate bait region, *tabula rasa* S1, were tested for their ability to improve the formation of native A2M and its inhibitory capacity toward MMP2. TR S1 QRT4 reintroduces the fourth quarter of the wild-type bait region. Two different S1 positions (with cleavage at position 710 or 703) were tested in TRΔ7, which shortens the TR bait region by seven residues. *B*, pore-limited native PAGE of A2Ms with the indicated bait regions. A2M TR S1 is expressed with a substantial amount of nonnative A2M. This nonnative A2M could be removed by depletion using LRP1-conjugated resin. Alternatively, the native content was improved in TRΔ7 and TR QRT4. *C*, the ability of the indicated A2Ms to inhibit MMP2’s digestion of DQ-gelatin was determined. Fitted curves calculated from the experimental data points by linear regression are shown as dotted lines. Error bars show the standard deviation and each sample was prepared in triplicate.

The initial *tabula rasa* A2M proteins were expressed with an increased amount of nonnative A2M compared with wild-type A2M. While this was not a major issue during their characterization in this study, as native A2M could be purified using LRP1-based depletion prior to inhibitory assays, such nonnative A2M constitutes a decrease in yield and its depletion would be difficult to scale up. In order to resolve this issue, we tested two altered *tabula rasa* bait regions (Fig. 6*A*). The first, TRΔ7, was shortened by seven residues to a total length of 32 residues, to compensate for the *tabula rasa* bait region possibly being effectively longer than the wild-type bait region due to altered dynamics, interactions, or secondary structure. The second, TR QRT4, reintroduced the C-terminal quarter of the wild-type bait region; while the bait region sequence in generally is not conserved, there is a tendency toward acidic and hydrophobic residues in its C-terminal quarter preceding the α’NT region (Fig. S1) and analogous residues in C3 are positioned alongside the MG3 domain (32), suggesting that this sequence may coordinate the position of the bait region. In fact, both TRΔ7 and TR QRT4 improved the native content to that of wild-type A2M (Fig. 6*B*). TRΔ7 is the preferable sequence for bait region design as it does not reintroduce any protease cleavage sites, in contrast to TR QRT4 (7).

### The position of the S1 substrate sequence within the TRΔ7 bait region affects the efficiency of MMP2 inhibition

MMP2 inhibition by A2M was assessed using the same fluorescent gelatin substrate used in the trypsin inhibition assays. While all *tabula rasa* A2Ms incorporating the S1 substrate site were capable of inhibiting MMP2, the bait region length and cleavage site position were found to affect the inhibitory efficiency (Fig. 6*C*). While the TRΔ7 S1 I703 bait region conveyed inhibition that was equivalent to that of plasma-purified or wild-type recombinant A2M, the TRΔ7 S1 I710 bait region, which was identical apart from the cleavage site position, inhibited with approximately 40% efficiency (Fig. 6*C*). The two 39-residue long *tabula rasa* bait regions TR S1 and TR S1 QRT4, which both incorporate the S1 cleavage site at position 710, had approximately 70% of the wild-type inhibitory capacity (Fig. 6*C*). These results demonstrate that *tabula rasa* A2Ms are capable of inhibiting MMP2 with the same efficiency as wild-type A2M, but that the bait region length and cleavage site position can affect the efficiency of protease inhibition and requires optimization.

## Discussion

In this study, we have produced and characterized 12 variant A2Ms in order to elucidate the effects of the *tabula rasa* bait region substitution and demonstrate an approach to developing new protease inhibitors, which selectively inhibit a target protease. Wild-type A2M is an efficient protease inhibitor with an extensive inhibitory repertoire, and it is well established that A2M can inhibit new proteases when an appropriate substrate sequence is added into the bait region (26, 27, 28). Furthermore, A2M is highly stable and has desirable pharmacokinetics, including a long circulatory half-life in its native conformation (12). Therefore, the main design challenge in developingA2M-based protease inhibitors is selectivity toward the target protease(s). We have shown that the *tabula rasa* bait region provides one solution to this issue and can advantageously be used as the starting point for bait region design, as it removes all known cleavage sites of human proteases from the A2M bait region while preserving A2M’s structure and protease-inhibiting functionality.

However, our investigation of *tabula rasa* bait regions incorporating MMP2 substrate sequences emphasizes a second challenge, which is the difficulty of finding substrate sequences that are truly specific to the target protease and will produce a completely selective A2M. Despite our testing of three distinct MMP2 motifs identified for MMP2 by phage display (47) as well as a generic MMP motif (Pro-X-X-hydrophobic), the resulting A2Ms were cleaved by all tested MMPs. Nonetheless, an A2M incorporating the S1 substrate was not cleaved by any of the tested non-MMP proteases, which is a significant improvement in specificity compared with wild-type A2M. A2M-based protease inhibition strategies may be aided in the future by the identification of more specific MMP substrate sequences, as our understanding of the relationship between MMP structure and substrate preference continues to improve (53). Of course, other promising approaches are under investigation that do not rely on distinct substrate preferences, such as the highly specific blocking of MMPs using monoclonal antibodies (54) or active site-directed nanobodies (55).

We have additionally shown how bait region factors affecting yield and inhibitory efficiency can be optimized. For example, the length of the *tabula rasa* bait region affected the formation of native A2M, and a shortened 32-residue *tabula rasa* bait region was preferable to the initial 39-residue *tabula rasa* bait region. The addition of a lysine residue into the *tabula rasa* bait region resulted in autoconjugation of the lysine by the thiol ester upon bait region cleavage. Although this autoconjugation event did not appear to compete with the conjugation of trypsin, as similar conjugation of fluorescent trypsin by wild-type A2M and A2M TR K704 was observed, it may be relevant in the inhibition of other proteases and should be considered. This becomes especially important considering that the inhibition of trypsin by *tabula rasa* A2Ms was dependent on covalent protease conjugation; this is in contrast to wild-type A2M, which has been shown to inhibit several proteases (including trypsin) independent of covalent conjugation (9, 56). We speculate that altered bait region dynamics may affect the accessibility of the bait region and allow proteases to cleave the substrate site without fully entering A2M’s internal cavity, in which case they cannot be trapped noncovalently. Alternatively, an increased distance between *tabula rasa* bait regions might lengthen the duration between successive bait region cleavage events and thereby slow the closing of the trap, permitting proteases to escape A2M after a single bait region cleavage event. The relevance of rapid successive bait region cleavage is supported by the difference in inhibitory capacities between A2Ms containing bait region arginines or lysines, considering that trypsin cleaves arginine substrates more rapidly than lysine substrates (57). This difference in inhibition between lysine- and arginine-containing A2Ms persisted even in the presence of BAPN, indicating that it is not a consequence of thiol ester autoconjugation to the bait region lysine.

Furthermore, the position of a cleavage site within the *tabula rasa* bait region was found to affect A2M’s inhibitory stoichiometry toward MMP2, as seen when comparing the TRΔ7 S1 I710 and TRΔ7 S1 I703 bait regions. This suggests that the *tabula rasa* bait region can be accessed from outside the A2M tetramer where the protease is not trapped upon A2M’s collapse, depending on the position of the cleavage site. It is unclear whether this is unique to the *tabula rasa* bait region or whether cleavage site position affects inhibition by wild-type A2M. The bait regions of wild-type A2M are sufficiently close to each other to allow disulfide formation (42), and they may interact with each other in a manner that is conducive to efficient protease trapping. Nonetheless, it was possible to identify cleavage site positions, which conveyed MMP2 inhibition by *tabula rasa* A2M that was equivalent to wild-type A2M, showing that this is a critical but solvable issue in bait region design.

The complete replacement of all bait region residues with Gly-Gly-Ser triplets without disruption of A2M’s proteolytically induced conformational change has mechanistic implications for A2M and the broader αM protein superfamily. It is currently unknown how bait region cleavage is sensed by A2M and triggers its conformational change. It has previously been proposed that the proteolytic removal of the anaphylactic domain (which corresponds to the bait region) in αM complement factors disrupts interactions between the anaphylactic, MG3, and MG8 domains, thus triggering the overall conformational change (36). Our results with *tabula rasa* A2M show that putative side-chain-dependent interactions of the bait region are neither required for the formation of native A2M or for inducing the conformational change. These observations make it improbable that the bait region participates in essential MG3/MG8-stabilizing interactions. This may reflect a difference between A2M and the αM complement factors, but we consider it more likely that the fundamental aspects of the proteolytically induced conformational change are conserved within the αM superfamily, as the ubiquity of this conformational change in the superfamily suggests that it arose early in an ancestral protein. If this assumption is correct, another less obvious proteolysis-sensing trigger mechanism remains to be found.

Proteases are important therapeutic targets in many diseases. As A2M is an efficient, irreversible, and broad-spectrum protease inhibitor, it could plausibly be used for protease-inhibiting interventions in many contexts. For example, both endogenous patient-enriched A2M and recombinant A2M are under investigation for clinical treatment of osteoarthritis (https://clinicaltrials.gov/ct2/show/NCT03656575) (58), where rampant cartilage degradation by MMPs is pathogenic. However, it is desirable to restrict these interventions to the target protease as much as possible, as off-target effects may be detrimental and/or dose-limiting and will obfuscate the on-target effects that are under clinical investigation. Here, we have shown that A2M tolerates complete replacement of its bait region and have demonstrated how this can be used in order to develop rationally designed protease inhibitors with restricted inhibitory profiles.

## Experimental procedures

### Gene design

A pcDNA3.1(+) plasmid with the gene for wild-type A2M under control of a CMV promoter (44) was used for recombinant expression of A2M. The bait region sequence was changed using either site-directed mutagenesis or synthesis of new bait region sequences followed by restriction site cloning, depending on the extent of the changes. All cloning work was performed by GenScript. All bait region sequences are shown in Table S1, and all full protein sequences are included in the Supplementary information.

Another pcDNA3.1(+) plasmid encoding human proMMP2 with two StrepII tags at the N-terminal end of its activation peptide was prepared by gene synthesis and cloning into pcDNA3.1(+), by Genscript.

### Expression and purification of A2M

All recombinant A2Ms were expressed in HEK293 FreeStyle cells using a standard transient transfection protocol. Briefly, 25 kDa linear polyethyleneimine (Polysciences) and plasmid DNA were incubated for 10 min in antibiotic-free FreeStyle medium (Thermo Fisher Scientific) at a 4:1 w/w PEI:DNA ratio, then slowly dripped into a culture of cells at a density of 1 million cells per ml, to a final DNA concentration of 1 μg per ml culture. After 4 days, the supernatant was harvested by spinning down the cells at 1500*g* and adding pH 7.4 HEPES to a final concentration of 50 mM.

Purification of recombinant A2M was performed using an established protocol (16, 59). Supernatants were first run through a Zn^2+^-loaded Chelating HiTrap column (GE Healthcare) and eluted with 50 mM EDTA, 150 mM NaCl, 100 mM sodium acetate, pH 7.4. The EDTA eluate was dialyzed against 20 mM HEPES at pH 7.4, then loaded onto a HiTrap Q column (GE Healthcare), and eluted by a gradient of 0 to 400 mM NaCl (with a constant 20 mM HEPES at pH 7.4). Fractions containing A2M were pooled, concentrated by ultrafiltration, and purified by size-exclusion chromatography on a Sephacryl S-300 HR (GE Healthcare), using a 20 mM HEPES, 150 mM NaCl, pH 7.4 running buffer (HEPES-buffered saline, HBS). Endogenous human A2M was purified from plasma provided by a healthy volunteer using this same protocol; prior to the first chromatography step, 4% polyethylene glycol (PEG) was used to precipitate and remove contaminant plasma proteins, after which 16% PEG was used to precipitate and isolate A2M for further purification.

### Protease production

N-terminally StrepII-tagged proMMP2 was expressed using the same transient transfection protocol as A2M. Supernatants were treated with streptavidin to remove free biotin. ProMMP2 was then purified by StrepTactin affinity chromatography (IBA Lifesciences), followed by size-exclusion chromatography on a Superdex 200 Increase (GE Healthcare). ProMMP2 was activated using 1 mM APMA by incubating for 15 min at 37 °C, followed by desalting into HBS with 10 mM CaCl_2_ using a PD-10 column (GE Healthcare).

Recombinant expression, purification, and activation of MMP-1, -3, -8, and -13, as well as ADAMTS-4 lacking the C-terminal spacer domain and ADAMTS-5 lacking the C-terminal thrombospondin domain, were performed as previously described (60, 61, 62, 63, 64, 65). Plasminogen was purified from human plasma as previously described (66) and activated by adding urokinase to a 1:100 w/w ratio and incubating at 37 °C for 2 h. Human neutrophil cathepsin G was purchased from Athens Research & Technology, bovine pancreatic trypsin from Sigma Aldrich, and lysyl endopeptidase (LysC) from FUJIFILM Wako Pure Chemical Corporation.

### Reaction of A2M with methylamine and proteases

To aminolyze A2M’s thiol ester, methylamine (pH 8) was added to 250 mM and incubated for at least 45 min at 37 °C. To assess the cleavage of A2M by trypsin and LysC, proteases were added to a 2.2:1 mol/mol ratio of protease:A2M and incubated for 5 min at 37 °C. The digestion was then inhibited using the serine protease inhibitor PMSF (2 mM, 15 min, room temperature). To assess the cleavage of A2M by MMP2, MMP2 was added to A2M in HBS with 10 mM CaCl_2_ to a 6:1 mol/mol ratio of MMP2:A2M, incubated for 15 min at 37 °C, and then inhibited using 20 mM EDTA. When cleaving A2M using other human proteases, incubation lasted 1 h at 37 °C in HBS with 10 mM CaCl_2_, and PMSF or EDTA was used to inhibit serine proteases and metalloproteases, respectively.

### In-gel digest using pepsin and LC-MS/MS analysis

The suspected autoconjugation product observed upon proteolytic activation of A2M TR K704 mutant was investigated by digesting the autoconjugation product separated by reducing SDS-PAGE with pepsin and subsequent LC-MS/MS analysis. Gel bands were excised, shrunk with acetonitrile, and then swelled with 0.1% v/v acetic acid, pH 3. Shrinking and swelling were repeated twice to wash the gel bands. The gel bands were shrunk a final time, dried, and then swelled in 0.1% v/v acetic acid, pH 3 with pepsin added to a final 1:20 w/w ratio of pepsin:sample. Digestion with pepsin was carried out overnight at 37 °C. Peptides from the digested samples were then purified using pipette tips packed with POROS 50 R2 C18 resin (PerSeptive Biosystems).

Approximately 250 ng of peptide was analyzed by LC-MS/MS with an EASY-nLC 1200 (Thermo Fisher Scientific) and an Orbitrap Eclipse Tribrid mass spectrometer (Thermo Fisher Scientific). A data-dependent acquisition method selected peptides for fragmentation by high-energy collision dissociation (HCD) and MS2; upon detection of the b2 and b3 fragment ions from the thiol-ester-covering LQMPYGCGEQN peptide, precursors were selected for a second MS2 scan using electron transfer dissociation (ETD), which resulted in the flagging of relevant precursor peptides with ETD scans.

Cross-linked peptide identification was performed manually. The most abundant precursor whose HCD MS2 spectrum triggered ETD and therefore contained LQMPYGCGEQN’s b2 and b3 fragment ions had a mass corresponding to the LQMPYGCGEQN and FTNSKIRKPKMCGGSGGSGGSGGSGGK peptides cross-linked by an isopeptide bond. Both cysteines were propionamidylated as they had been reduced prior to SDS-PAGE; there were no other amino acid modifications. Fragment ions in this precursor’s HCD MS2 spectrum were manually annotated with a mass tolerance of 10 ppm. Manual annotation identified ions resulting from fragmentation in both peptides, in addition to *b*- and *y*-type single-fragmented ions. The raw data file containing the annotated spectrum has been deposited to the ProteomeXchange Consortium *via* the PRIDE (67) partner repository with the dataset identifier PXD023651.

### Depletion of nonnative A2M using LRP1-conjugated resin

The A2M-binding fragment of LRP1, cluster 1B (68), was expressed with two N-terminal StrepII tags and a C-terminal Fc region from human IgG1 and purified as previously described (11). In total, 600 μg LRP1 was conjugated onto 200 mg of NHS-activated agarose (Pierce) in 0.15 M TEAB, 0.15 M HEPES, pH 8.3, for 2 h at room temperature with mixing on a rotator. Conjugation was quenched with 50 mM Tris-HCl, pH 8.

Nonnative recombinant A2M was depleted by adding 10 mM CaCl_2_ to the A2M solution and incubating it for at least 2 h with the LRP1 resin on a rotator at room temperature. The flowthrough from the resin was then saved as the depleted A2M sample, and the resin was regenerated first by eluting with three rounds of 50 mM EDTA in HBS, followed by three rounds of washing with 10 mM CaCl_2_ in HBS. The abundance of native A2M in the sample before and after depletion was then assessed by pore-limited native PAGE.

### Determining A2M’s inhibition of protein substrate cleavage by trypsin and MMP2

The inhibition of trypsin by A2M was investigated using a fluorescently labeled gelatin substrate. In total, 2.1 pmol (11.9 nM) of active trypsin was reacted with 0 to 8.6 pmol (0–47.7 nM) of A2M in 50 mM HEPES, 100 mM NaCl, 5 mM CaCl_2_, pH 8 for 15 min at 37 °C. DQ gelatin from pig skin (Invitrogen) was added to a final concentration of 0.1 mg/ml. The fluorescence (excitation at 485 nm and emission at 520 nm) of the unquenched digestion products of DQ gelatin after 2 min at 37 °C was measured in a FLUOstar Omega plate reader (BMG LABTECH). Where noted, 50 mM BAPN was included in the initial reaction buffer with trypsin and A2M. All reactions were performed in triplicates.

Similar reactions were carried out to determine the inhibition of MMP2 by recombinant wild-type A2M and the mutants TR+ S1, TRd7 S1, TR S1, and TR d7 I703. The only difference in the experimental setup was that 1.4 pmol (7.5 nM) of MMP2 was reacted with 0 to 2.7 pmol (0–15 nM) of A2M, and fluorescence was measured after 10 min of incubating the gelatin with the A2M/MMP2 reactions.

## Data availability

The raw data file containing the spectrum identifying A2M TR K704’s autoconjugation product peptide has been deposited to the ProteomeXchange Consortium *via* the PRIDE (67) partner repository with the dataset identifier PXD023651.

Full protein sequences for all recombinant A2M proteins are included in the supplementary information.

## Supporting information

This article contains supporting information.

## Conflict of interest

The authors declare that they have no conflicts of interest with the contents of this article.
