# Supplementary material for: Development of selective protease inhibitors via engineering of the bait region of human α2-macroglobulin
Source: J Biol Chem. 2021 Jun 15;297(1):100879. doi: 10.1016/j.jbc.2021.100879 (PMC8267569; doi:10.1016/j.jbc.2021.100879)
Supplement: Supplemental Figures S1–S6 and Table S1 [file mmc1.docx]

**Supplementary information**

**Development of selective protease inhibitors via engineering of the bait region of human α_2_-macroglobulin**

Seandean Lykke Harwood^1,2^, Nadia Sukusu Nielsen^1^, Khang Diep^1^, Kathrine Tejlgård Jensen^1^,

Peter Kresten Nielsen^2^, Kazuhiro Yamamoto^3^, Jan J. Enghild^1*^

^1^ Department of Molecular Biology and Genetics, Aarhus University, Aarhus 8000, Denmark

^2^ Global Research Technologies, Novo Nordisk A/S, Novo Nordisk Park, Måløv 2760, Denmark

^3^ Institute of Ageing and Chronic Disease, University of Liverpool, Liverpool L7 8TX, United Kingdom

**Supporting information in this document**

[Table S1. Bait region sequences of recombinant A2M proteins. S-2](#_Toc61973621)

[FIGURE S1. Alignment of bait region sequences from orthologous vertebrate A2Ms. S-3](#_Toc61973622)

[FIGURE S2. SEC investigating tetramerization of *tabula rasa* A2M. S-4](#_Toc61973623)

[FIGURE S3. Depletion of non-native A2M using LRP1-conjugated resin S-5](#_Toc61973625)

[FIGURE S4. Characterization of additional lysine- and arginine-containing bait regions. S-6](#_Toc61973624)

[FIGURE S5. Characterization of A2M:MMP2 complex formation. S-7](#_Toc61973626)

[FIGURE S6. Screening *tabula rasa* bait regions with MMP2 substrates sequences S-8](#_Toc61973627)

[Full sequences for all recombinant A2M proteins S-9](#_Toc61973631)

**Other supporting information:**

**MS data:** The raw mass spectrometry data file used to identify the cross-linked peptide showing auto-conjugation of Lys704 for the A2M TR K704 protein has been deposited to the ProteomeXchange Consortium via the PRIDE partner repository^68^ with the dataset identifier PXD023651.

## SUPPLEMENTARY TABLE S1. Bait region sequences of recombinant A2M proteins.

The sequences of all A2M mutants used in this study are given between the Cys689 and Phe735 residues (numbered according to wildtype A2M), which covers all differences. Cleavage sites are bolded.

| Name | Sequence from Cys689 to F735 |
| --- | --- |
| Wildtype | CPQLQQYEMHGPEGLRVGFYESDVMGRGHARLVHVEEPHTETVRKYF |
| TR | CGGSGGSGGSGGSGGSGGSGGSGGSGGSGGSGGSGGSGGSETVRKYF |
| TR K704 | CGGSGGSGGSGGSGG**K**GGSGGSGGSGGSGGSGGSGGSGGSETVRKYF |
| TR R704 | CGGSGGSGGSGGSGG**R**GGSGGSGGSGGSGGSGGSGGSGGSETVRKYF |
| TR K710 | CGGSGGSGGSGGSGGSGGSGG**K**GGSGGSGGSGGSGGSGGSETVRKYF |
| TR R710 | CGGSGGSGGSGGSGGSGGSGG**R**GGSGGSGGSGGSGGSGGSETVRKYF |
| TR A21A | CGGSGGSGGSGGSGGSG**HMHAALTA**GGSGGSGGSGGSGGSETVRKYF |
| TR B74 | CGGSGGSGGSGGSGGSG**ESLAYYTA**GGSGGSGGSGGSGGSETVRKYF |
| TR C9 | CGGSGGSGGSGGSGGSG**RSLSRLTA**GGSGGSGGSGGSGGSETVRKYF |
| TR S1 | CGGSGGSGGSGGSGGGGS**PQGIAGQ**GGSGGSGGSGGSGGSETVRKYF |
| TR S1 QRT4 | CGGSGGSGGSGGSGGGGS**PQGIAGQ**GGSGGLVHVEEPHTETVRKYF |
| TR∆7 S1 I710 | CGGSGGSGGSGGSGGGGS**PQGIAGQ**GGSGGSGGETVRKYF |
| TR∆7 S1 I703 | CGGSGGSGGSG**PQGIAGQ**SGGSGGSGGSGGSGGETVRKYF |


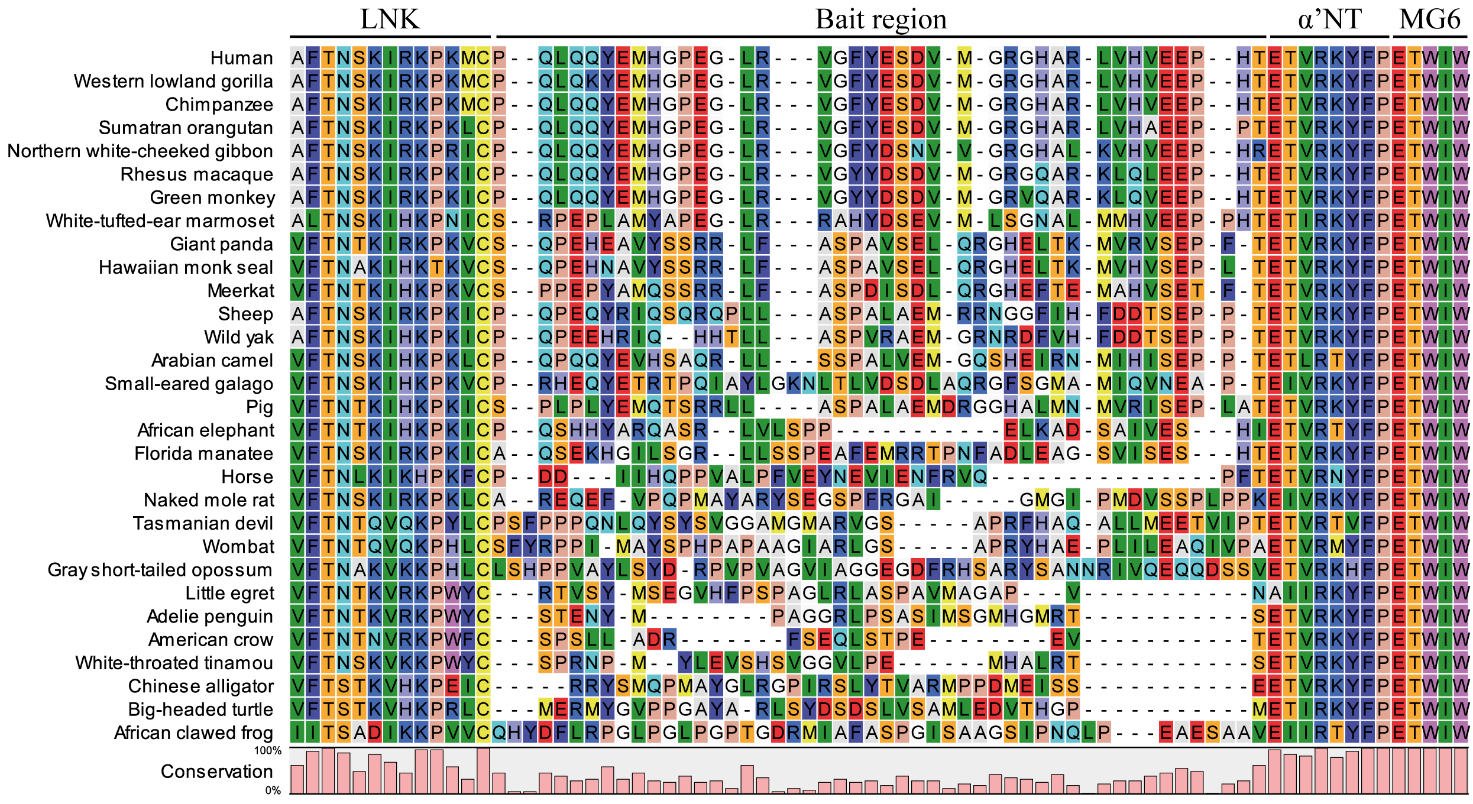


## SUPPLEMENTARY FIGURE S1. Alignment of bait region sequences from orthologous vertebrate A2Ms. Vertebrate A2M protein sequences with amino acid lengths between 1400 and 1500 were found on UniProt and aligned using CLC Workbench (Qiagen). The shown sequence spans from residues 677 to 741 in human A2M. The bait region is flanked by the LNK region, the α’NT sequence, and the MG6 domain which are highly conserved across vertebrates, but sequence conservation within the bait region is low. There are no obvious conserved motifs in the bait region, although most have diverse amino acid compositions in general, and there is often a mix of hydrophobic and acidic residues towards the C-terminal end.


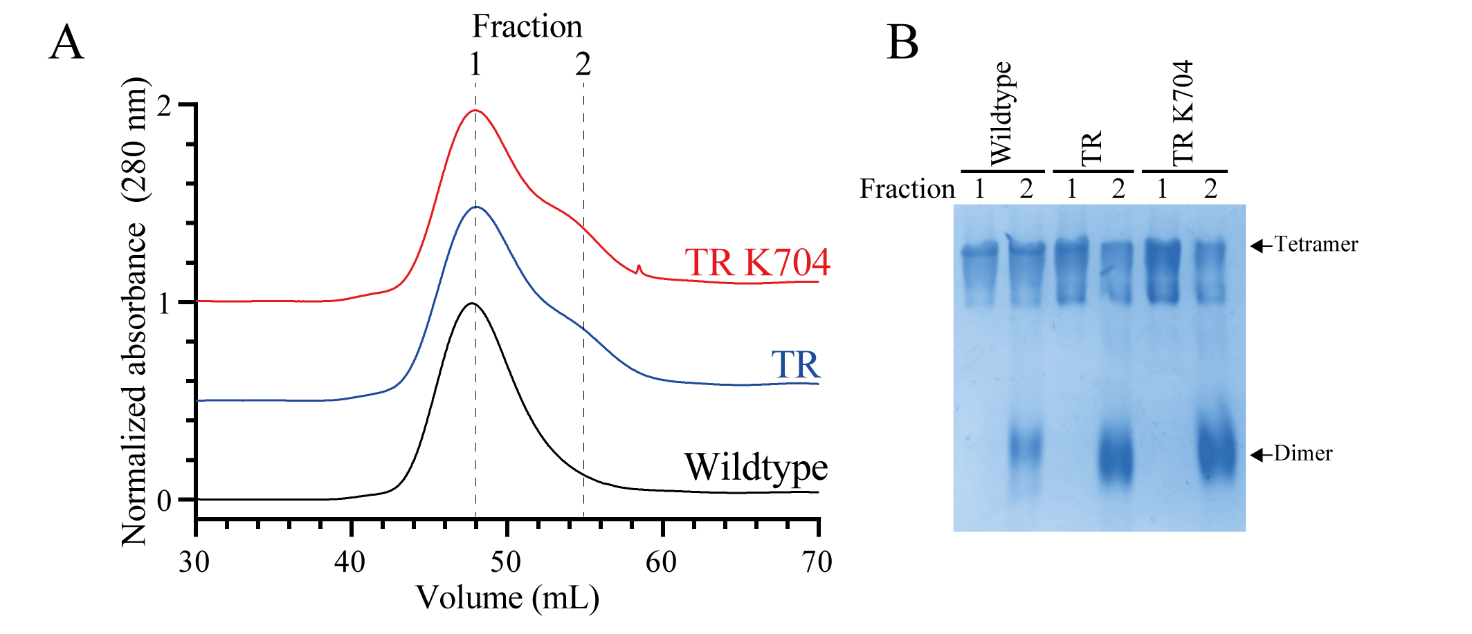


## SUPPLEMENTARY FIGURE S2. SEC investigating tetramerization of *tabula rasa* A2M. (A) Recombinant wildtype A2M, A2M TR, and A2M TR K704 were separated by SEC on a Sephacryl S-300 HR (GE Healthcare), following their purification by Zn^2+^ affinity and ion exchange chromatography. Both *tabula rasa* A2M elution peaks had a more prominent shoulder following the main peak than wildtype A2M. (B) The indicated fractions from panel A were investigated by pore limited native PAGE. This showed that the shoulder corresponds to dimeric A2M, indicating that a higher proportion of *tabula rasa* A2M is produced as dimers, although the majority does still form tetramers.


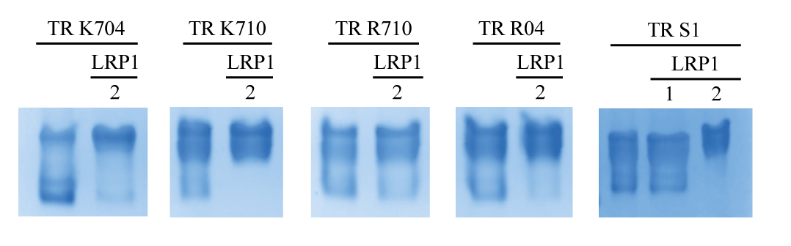


**SUPPLEMENTARY** FIGURE S3. **Depletion of non-native A2M using LRP1-conjugated resin.** Recombinant A2Ms with significant non-native A2M content were depleted using LRP1-conjugated resin in order to purify native, functional A2M prior to quantitative determination of their inhibitory capacity towards proteases. The A2M samples before depletion and after the indicated number of rounds of LRP1 depletion were analyzed by pore limited native PAGE to verify that non-native A2M had been removed.


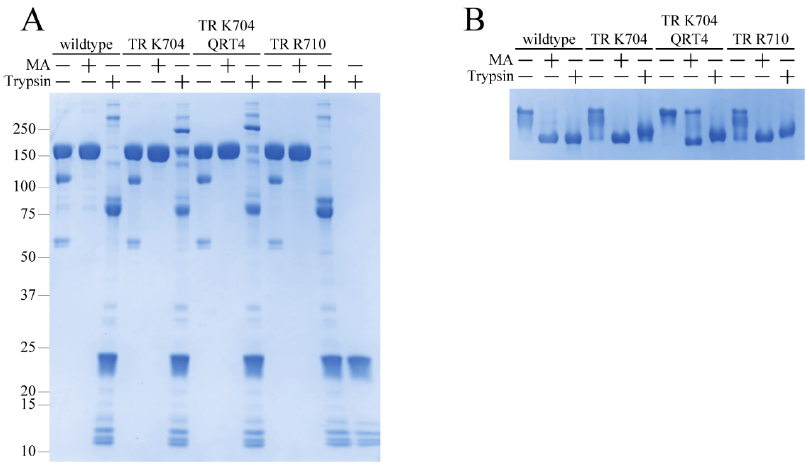
SUPPLEMENTARY FIGURE S4. **Characterization of additional lysine- and arginine-containing *tabula rasa* A2Ms**. Reducing SDS-PAGE (**A**) and pore limited native PAGE (**B**) of additional lysine- and arginine-containing tabula rasa bait regions, treated with methylamine and trypsin to demonstrate their aminolysis- and proteolysis-induced conformational changes.


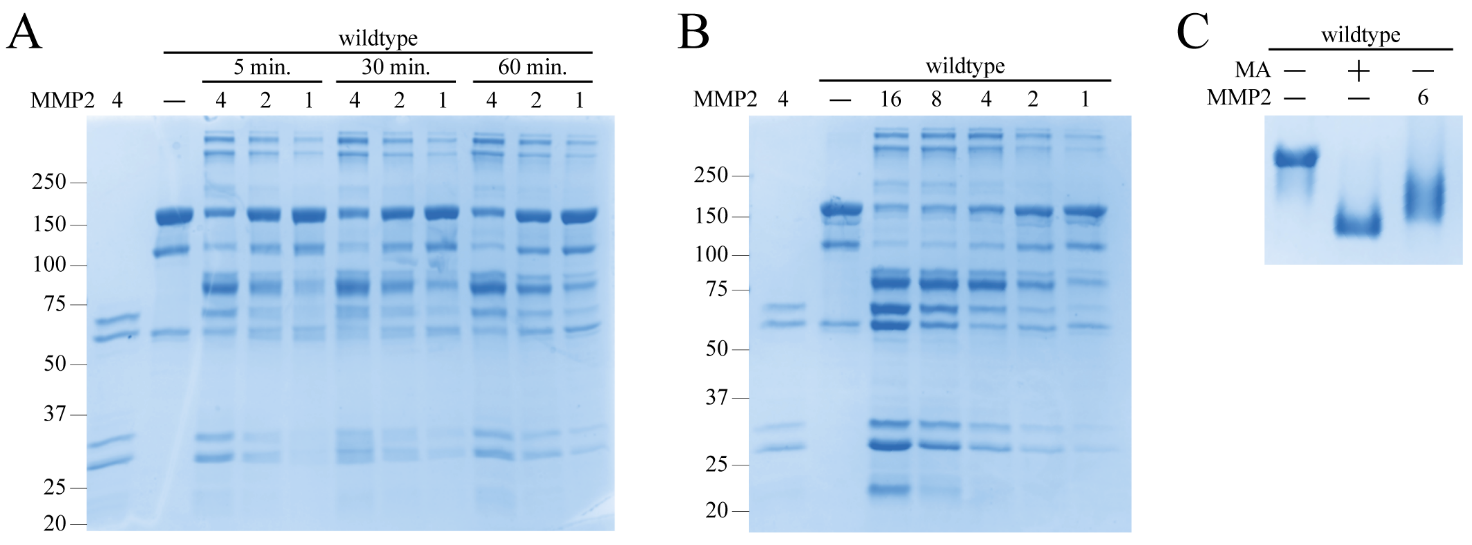


## SUPPLEMENTARY FIGURE S5. Characterization of A2M:MMP2 complex formation.

(**A**) Wildtype A2M was digested by the indicated mol:mol ratios of MMP2:A2M for 5, 30, or 60 minutes., after which MMP2 was inhibited by EDTA. Samples were then analyzed by reducing SDS-PAGE. The digestion of A2M by MMP2 is complete after 5 minutes. (**B**) Wildtype A2M was digested for 15 minutes by the indicated molar ratios of MMP2. While complete A2M digestion was never observed, more digestion was not observed at 16:1 compared to 8:1 ratios. A2M’s digestion was at its maximum between 4:1 and 8:1 ratios, and a 6:1 ratio was therefore used in later experiments. (**C**) Pore limited native PAGE of wildtype A2M with methylamine or MMP2 treatment. The A2M-MMP2 complex has an intermediate electrophoretic mobility compared to the methylamine-treated conformation, in contrast to most proteases (e.g. trypsin) whose complexes with A2M show the same high electrophoretic mobility.


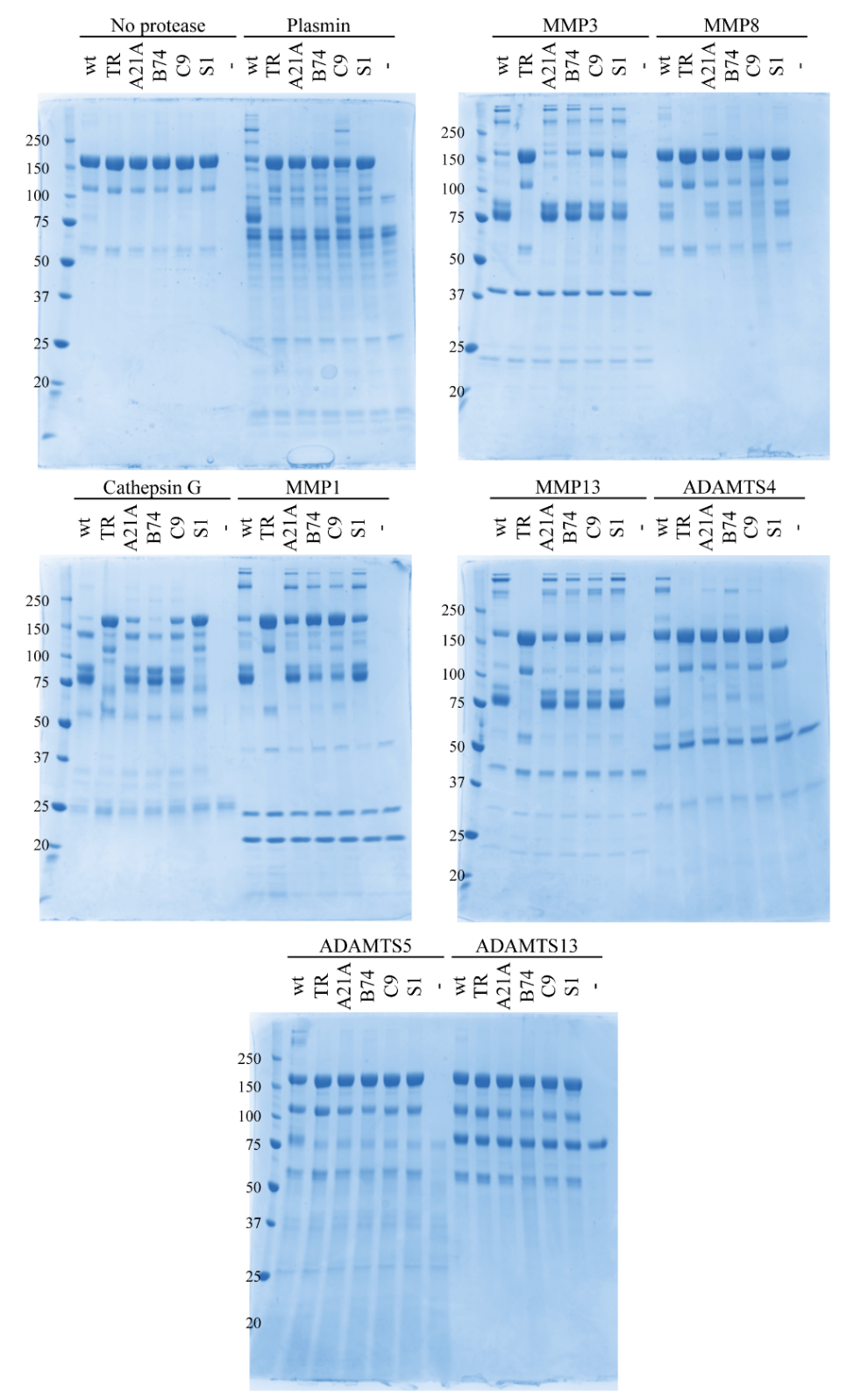


SUPPLEMENTARY FIGURE S6. **Screening *tabula rasa* bait regions with MMP2 substrates sequences using an additional nine human proteases.** Wildtype A2M and *tabula rasa* A2M with no protease cleavage site or with one of four MMP2 substrate sites (A21A, B74, C9, S1) were digested by nine human proteases and analyzed by reducing SDS-PAGE. Bait region cleavage is apparent by the appearance of the N-terminal and C-terminal bait region cleavage fragments and/or by the appearance of high-MW multimeric thiol ester conjugation products.

## Full sequences for all recombinant A2M proteins

>A2M_wildtype

MGKNKLLHPSLVLLLLVLLPTDASVSGKPQYMVLVPSLLHTETTEKGCVLLSYLNETVTVSASLESVRGNRSLFTDLEAENDVLHCVAFAVPKSSSNEEVMFLTVQVKGPTQEFKKRTTVMVKNEDSLVFVQTDKSIYKPGQTVKFRVVSMDENFHPLNELIPLVYIQDPKGNRIAQWQSFQLEGGLKQFSFPLSSEPFQGSYKVVVQKKSGGRTEHPFTVEEFVLPKFEVQVTVPKIITILEEEMNVSVCGLYTYGKPVPGHVTVSICRKYSDASDCHGEDSQAFCEKFSGQLNSHGCFYQQVKTKVFQLKRKEYEMKLHTEAQIQEEGTVVELTGRQSSEITRTITKLSFVKVDSHFRQGIPFFGQVRLVDGKGVPIPNKVIFIRGNEANYYSNATTDEHGLVQFSINTTNVMGTSLTVRVNYKDRSPCYGYQWVSEEHEEAHHTAYLVFSPSKSFVHLEPMSHELPCGHTQTVQAHYILNGGTLLGLKKLSFYYLIMAKGGIVRTGTHGLLVKQEDMKGHFSISIPVKSDIAPVARLLIYAVLPTGDVIGDSAKYDVENCLANKVDLSFSPSQSLPASHAHLRVTAAPQSVCALRAVDQSVLLMKPDAELSASSVYNLLPEKDLTGFPGPLNDQDDEDCINRHNVYINGITYTPVSSTNEKDMYSFLEDMGLKAFTNSKIRKPKMCPQLQQYEMHGPEGLRVGFYESDVMGRGHARLVHVEEPHTETVRKYFPETWIWDLVVVNSAGVAEVGVTVPDTITEWKAGAFCLSEDAGLGISSTASLRAFQPFFVELTMPYSVIRGEAFTLKATVLNYLPKCIRVSVQLEASPAFLAVPVEKEQAPHCICANGRQTVSWAVTPKSLGNVNFTVSAEALESQELCGTEVPSVPEHGRKDTVIKPLLVEPEGLEKETTFNSLLCPSGGEVSEELSLKLPPNVVEESARASVSVLGDILGSAMQNTQNLLQMPYGCGEQNMVLFAPNIYVLDYLNETQQLTPEVKSKAIGYLNTGYQRQLNYKHYDGSYSTFGERYGRNQGNTWLTAFVLKTFAQARAYIFIDEAHITQALIWLSQRQKDNGCFRSSGSLLNNAIKGGVEDEVTLSAYITIALLEIPLTVTHPVVRNALFCLESAWKTAQEGDHGSHVYTKALLAYAFALAGNQDKRKEVLKSLNEEAVKKDNSVHWERPQKPKAPVGHFYEPQAPSAEVEMTSYVLLAYLTAQPAPTSEDLTSATNIVKWITKQQNAQGGFSSTQDTVVALHALSKYGAATFTRTGKAAQVTIQSSGTFSSKFQVDNNNRLLLQQVSLPELPGEYSMKVTGEGCVYLQTSLKYNILPEKEEFPFALGVQTLPQTCDEPKAHTSFQISLSVSYTGSRSASNMAIVDVKMVSGFIPLKPTVKMLERSNHVSRTEVSSNHVLIYLDKVSNQTLSLFFTVLQDVPVRDLKPAIVKVYDYYETDEFAIAEYNAPCSKDLGNA*

>A2M_TR

MGKNKLLHPSLVLLLLVLLPTDASVSGKPQYMVLVPSLLHTETTEKGCVLLSYLNETVTVSASLESVRGNRSLFTDLEAENDVLHCVAFAVPKSSSNEEVMFLTVQVKGPTQEFKKRTTVMVKNEDSLVFVQTDKSIYKPGQTVKFRVVSMDENFHPLNELIPLVYIQDPKGNRIAQWQSFQLEGGLKQFSFPLSSEPFQGSYKVVVQKKSGGRTEHPFTVEEFVLPKFEVQVTVPKIITILEEEMNVSVCGLYTYGKPVPGHVTVSICRKYSDASDCHGEDSQAFCEKFSGQLNSHGCFYQQVKTKVFQLKRKEYEMKLHTEAQIQEEGTVVELTGRQSSEITRTITKLSFVKVDSHFRQGIPFFGQVRLVDGKGVPIPNKVIFIRGNEANYYSNATTDEHGLVQFSINTTNVMGTSLTVRVNYKDRSPCYGYQWVSEEHEEAHHTAYLVFSPSKSFVHLEPMSHELPCGHTQTVQAHYILNGGTLLGLKKLSFYYLIMAKGGIVRTGTHGLLVKQEDMKGHFSISIPVKSDIAPVARLLIYAVLPTGDVIGDSAKYDVENCLANKVDLSFSPSQSLPASHAHLRVTAAPQSVCALRAVDQSVLLMKPDAELSASSVYNLLPEKDLTGFPGPLNDQDDEDCINRHNVYINGITYTPVSSTNEKDMYSFLEDMGLKAFTNSKIRKPKMCGGSGGSGGSGGSGGSGGSGGSGGSGGSGGSGGSGGSGGSETVRKYFPETWIWDLVVVNSAGVAEVGVTVPDTITEWKAGAFCLSEDAGLGISSTASLRAFQPFFVELTMPYSVIRGEAFTLKATVLNYLPKCIRVSVQLEASPAFLAVPVEKEQAPHCICANGRQTVSWAVTPKSLGNVNFTVSAEALESQELCGTEVPSVPEHGRKDTVIKPLLVEPEGLEKETTFNSLLCPSGGEVSEELSLKLPPNVVEESARASVSVLGDILGSAMQNTQNLLQMPYGCGEQNMVLFAPNIYVLDYLNETQQLTPEVKSKAIGYLNTGYQRQLNYKHYDGSYSTFGERYGRNQGNTWLTAFVLKTFAQARAYIFIDEAHITQALIWLSQRQKDNGCFRSSGSLLNNAIKGGVEDEVTLSAYITIALLEIPLTVTHPVVRNALFCLESAWKTAQEGDHGSHVYTKALLAYAFALAGNQDKRKEVLKSLNEEAVKKDNSVHWERPQKPKAPVGHFYEPQAPSAEVEMTSYVLLAYLTAQPAPTSEDLTSATNIVKWITKQQNAQGGFSSTQDTVVALHALSKYGAATFTRTGKAAQVTIQSSGTFSSKFQVDNNNRLLLQQVSLPELPGEYSMKVTGEGCVYLQTSLKYNILPEKEEFPFALGVQTLPQTCDEPKAHTSFQISLSVSYTGSRSASNMAIVDVKMVSGFIPLKPTVKMLERSNHVSRTEVSSNHVLIYLDKVSNQTLSLFFTVLQDVPVRDLKPAIVKVYDYYETDEFAIAEYNAPCSKDLGNA*

>A2M_TR_K704

MGKNKLLHPSLVLLLLVLLPTDASVSGKPQYMVLVPSLLHTETTEKGCVLLSYLNETVTVSASLESVRGNRSLFTDLEAENDVLHCVAFAVPKSSSNEEVMFLTVQVKGPTQEFKKRTTVMVKNEDSLVFVQTDKSIYKPGQTVKFRVVSMDENFHPLNELIPLVYIQDPKGNRIAQWQSFQLEGGLKQFSFPLSSEPFQGSYKVVVQKKSGGRTEHPFTVEEFVLPKFEVQVTVPKIITILEEEMNVSVCGLYTYGKPVPGHVTVSICRKYSDASDCHGEDSQAFCEKFSGQLNSHGCFYQQVKTKVFQLKRKEYEMKLHTEAQIQEEGTVVELTGRQSSEITRTITKLSFVKVDSHFRQGIPFFGQVRLVDGKGVPIPNKVIFIRGNEANYYSNATTDEHGLVQFSINTTNVMGTSLTVRVNYKDRSPCYGYQWVSEEHEEAHHTAYLVFSPSKSFVHLEPMSHELPCGHTQTVQAHYILNGGTLLGLKKLSFYYLIMAKGGIVRTGTHGLLVKQEDMKGHFSISIPVKSDIAPVARLLIYAVLPTGDVIGDSAKYDVENCLANKVDLSFSPSQSLPASHAHLRVTAAPQSVCALRAVDQSVLLMKPDAELSASSVYNLLPEKDLTGFPGPLNDQDDEDCINRHNVYINGITYTPVSSTNEKDMYSFLEDMGLKAFTNSKIRKPKMCGGSGGSGGSGGSGGKGGSGGSGGSGGSGGSGGSGGSGGSETVRKYFPETWIWDLVVVNSAGVAEVGVTVPDTITEWKAGAFCLSEDAGLGISSTASLRAFQPFFVELTMPYSVIRGEAFTLKATVLNYLPKCIRVSVQLEASPAFLAVPVEKEQAPHCICANGRQTVSWAVTPKSLGNVNFTVSAEALESQELCGTEVPSVPEHGRKDTVIKPLLVEPEGLEKETTFNSLLCPSGGEVSEELSLKLPPNVVEESARASVSVLGDILGSAMQNTQNLLQMPYGCGEQNMVLFAPNIYVLDYLNETQQLTPEVKSKAIGYLNTGYQRQLNYKHYDGSYSTFGERYGRNQGNTWLTAFVLKTFAQARAYIFIDEAHITQALIWLSQRQKDNGCFRSSGSLLNNAIKGGVEDEVTLSAYITIALLEIPLTVTHPVVRNALFCLESAWKTAQEGDHGSHVYTKALLAYAFALAGNQDKRKEVLKSLNEEAVKKDNSVHWERPQKPKAPVGHFYEPQAPSAEVEMTSYVLLAYLTAQPAPTSEDLTSATNIVKWITKQQNAQGGFSSTQDTVVALHALSKYGAATFTRTGKAAQVTIQSSGTFSSKFQVDNNNRLLLQQVSLPELPGEYSMKVTGEGCVYLQTSLKYNILPEKEEFPFALGVQTLPQTCDEPKAHTSFQISLSVSYTGSRSASNMAIVDVKMVSGFIPLKPTVKMLERSNHVSRTEVSSNHVLIYLDKVSNQTLSLFFTVLQDVPVRDLKPAIVKVYDYYETDEFAIAEYNAPCSKDLGNA*

>A2M_TR_R710

MGKNKLLHPSLVLLLLVLLPTDASVSGKPQYMVLVPSLLHTETTEKGCVLLSYLNETVTVSASLESVRGNRSLFTDLEAENDVLHCVAFAVPKSSSNEEVMFLTVQVKGPTQEFKKRTTVMVKNEDSLVFVQTDKSIYKPGQTVKFRVVSMDENFHPLNELIPLVYIQDPKGNRIAQWQSFQLEGGLKQFSFPLSSEPFQGSYKVVVQKKSGGRTEHPFTVEEFVLPKFEVQVTVPKIITILEEEMNVSVCGLYTYGKPVPGHVTVSICRKYSDASDCHGEDSQAFCEKFSGQLNSHGCFYQQVKTKVFQLKRKEYEMKLHTEAQIQEEGTVVELTGRQSSEITRTITKLSFVKVDSHFRQGIPFFGQVRLVDGKGVPIPNKVIFIRGNEANYYSNATTDEHGLVQFSINTTNVMGTSLTVRVNYKDRSPCYGYQWVSEEHEEAHHTAYLVFSPSKSFVHLEPMSHELPCGHTQTVQAHYILNGGTLLGLKKLSFYYLIMAKGGIVRTGTHGLLVKQEDMKGHFSISIPVKSDIAPVARLLIYAVLPTGDVIGDSAKYDVENCLANKVDLSFSPSQSLPASHAHLRVTAAPQSVCALRAVDQSVLLMKPDAELSASSVYNLLPEKDLTGFPGPLNDQDDEDCINRHNVYINGITYTPVSSTNEKDMYSFLEDMGLKAFTNSKIRKPKMCGGSGGSGGSGGSGGSGGSGGRGGSGGSGGSGGSGGSGGSETVRKYFPETWIWDLVVVNSAGVAEVGVTVPDTITEWKAGAFCLSEDAGLGISSTASLRAFQPFFVELTMPYSVIRGEAFTLKATVLNYLPKCIRVSVQLEASPAFLAVPVEKEQAPHCICANGRQTVSWAVTPKSLGNVNFTVSAEALESQELCGTEVPSVPEHGRKDTVIKPLLVEPEGLEKETTFNSLLCPSGGEVSEELSLKLPPNVVEESARASVSVLGDILGSAMQNTQNLLQMPYGCGEQNMVLFAPNIYVLDYLNETQQLTPEVKSKAIGYLNTGYQRQLNYKHYDGSYSTFGERYGRNQGNTWLTAFVLKTFAQARAYIFIDEAHITQALIWLSQRQKDNGCFRSSGSLLNNAIKGGVEDEVTLSAYITIALLEIPLTVTHPVVRNALFCLESAWKTAQEGDHGSHVYTKALLAYAFALAGNQDKRKEVLKSLNEEAVKKDNSVHWERPQKPKAPVGHFYEPQAPSAEVEMTSYVLLAYLTAQPAPTSEDLTSATNIVKWITKQQNAQGGFSSTQDTVVALHALSKYGAATFTRTGKAAQVTIQSSGTFSSKFQVDNNNRLLLQQVSLPELPGEYSMKVTGEGCVYLQTSLKYNILPEKEEFPFALGVQTLPQTCDEPKAHTSFQISLSVSYTGSRSASNMAIVDVKMVSGFIPLKPTVKMLERSNHVSRTEVSSNHVLIYLDKVSNQTLSLFFTVLQDVPVRDLKPAIVKVYDYYETDEFAIAEYNAPCSKDLGNA*

>A2M_TR_K710

MGKNKLLHPSLVLLLLVLLPTDASVSGKPQYMVLVPSLLHTETTEKGCVLLSYLNETVTVSASLESVRGNRSLFTDLEAENDVLHCVAFAVPKSSSNEEVMFLTVQVKGPTQEFKKRTTVMVKNEDSLVFVQTDKSIYKPGQTVKFRVVSMDENFHPLNELIPLVYIQDPKGNRIAQWQSFQLEGGLKQFSFPLSSEPFQGSYKVVVQKKSGGRTEHPFTVEEFVLPKFEVQVTVPKIITILEEEMNVSVCGLYTYGKPVPGHVTVSICRKYSDASDCHGEDSQAFCEKFSGQLNSHGCFYQQVKTKVFQLKRKEYEMKLHTEAQIQEEGTVVELTGRQSSEITRTITKLSFVKVDSHFRQGIPFFGQVRLVDGKGVPIPNKVIFIRGNEANYYSNATTDEHGLVQFSINTTNVMGTSLTVRVNYKDRSPCYGYQWVSEEHEEAHHTAYLVFSPSKSFVHLEPMSHELPCGHTQTVQAHYILNGGTLLGLKKLSFYYLIMAKGGIVRTGTHGLLVKQEDMKGHFSISIPVKSDIAPVARLLIYAVLPTGDVIGDSAKYDVENCLANKVDLSFSPSQSLPASHAHLRVTAAPQSVCALRAVDQSVLLMKPDAELSASSVYNLLPEKDLTGFPGPLNDQDDEDCINRHNVYINGITYTPVSSTNEKDMYSFLEDMGLKAFTNSKIRKPKMCGGSGGSGGSGGSGGSGGSGGKGGSGGSGGSGGSGGSGGSETVRKYFPETWIWDLVVVNSAGVAEVGVTVPDTITEWKAGAFCLSEDAGLGISSTASLRAFQPFFVELTMPYSVIRGEAFTLKATVLNYLPKCIRVSVQLEASPAFLAVPVEKEQAPHCICANGRQTVSWAVTPKSLGNVNFTVSAEALESQELCGTEVPSVPEHGRKDTVIKPLLVEPEGLEKETTFNSLLCPSGGEVSEELSLKLPPNVVEESARASVSVLGDILGSAMQNTQNLLQMPYGCGEQNMVLFAPNIYVLDYLNETQQLTPEVKSKAIGYLNTGYQRQLNYKHYDGSYSTFGERYGRNQGNTWLTAFVLKTFAQARAYIFIDEAHITQALIWLSQRQKDNGCFRSSGSLLNNAIKGGVEDEVTLSAYITIALLEIPLTVTHPVVRNALFCLESAWKTAQEGDHGSHVYTKALLAYAFALAGNQDKRKEVLKSLNEEAVKKDNSVHWERPQKPKAPVGHFYEPQAPSAEVEMTSYVLLAYLTAQPAPTSEDLTSATNIVKWITKQQNAQGGFSSTQDTVVALHALSKYGAATFTRTGKAAQVTIQSSGTFSSKFQVDNNNRLLLQQVSLPELPGEYSMKVTGEGCVYLQTSLKYNILPEKEEFPFALGVQTLPQTCDEPKAHTSFQISLSVSYTGSRSASNMAIVDVKMVSGFIPLKPTVKMLERSNHVSRTEVSSNHVLIYLDKVSNQTLSLFFTVLQDVPVRDLKPAIVKVYDYYETDEFAIAEYNAPCSKDLGNA*

>A2M_TR_R704

MGKNKLLHPSLVLLLLVLLPTDASVSGKPQYMVLVPSLLHTETTEKGCVLLSYLNETVTVSASLESVRGNRSLFTDLEAENDVLHCVAFAVPKSSSNEEVMFLTVQVKGPTQEFKKRTTVMVKNEDSLVFVQTDKSIYKPGQTVKFRVVSMDENFHPLNELIPLVYIQDPKGNRIAQWQSFQLEGGLKQFSFPLSSEPFQGSYKVVVQKKSGGRTEHPFTVEEFVLPKFEVQVTVPKIITILEEEMNVSVCGLYTYGKPVPGHVTVSICRKYSDASDCHGEDSQAFCEKFSGQLNSHGCFYQQVKTKVFQLKRKEYEMKLHTEAQIQEEGTVVELTGRQSSEITRTITKLSFVKVDSHFRQGIPFFGQVRLVDGKGVPIPNKVIFIRGNEANYYSNATTDEHGLVQFSINTTNVMGTSLTVRVNYKDRSPCYGYQWVSEEHEEAHHTAYLVFSPSKSFVHLEPMSHELPCGHTQTVQAHYILNGGTLLGLKKLSFYYLIMAKGGIVRTGTHGLLVKQEDMKGHFSISIPVKSDIAPVARLLIYAVLPTGDVIGDSAKYDVENCLANKVDLSFSPSQSLPASHAHLRVTAAPQSVCALRAVDQSVLLMKPDAELSASSVYNLLPEKDLTGFPGPLNDQDDEDCINRHNVYINGITYTPVSSTNEKDMYSFLEDMGLKAFTNSKIRKPKMCGGSGGSGGSGGSGGRGGSGGSGGSGGSGGSGGSGGSGGSETVRKYFPETWIWDLVVVNSAGVAEVGVTVPDTITEWKAGAFCLSEDAGLGISSTASLRAFQPFFVELTMPYSVIRGEAFTLKATVLNYLPKCIRVSVQLEASPAFLAVPVEKEQAPHCICANGRQTVSWAVTPKSLGNVNFTVSAEALESQELCGTEVPSVPEHGRKDTVIKPLLVEPEGLEKETTFNSLLCPSGGEVSEELSLKLPPNVVEESARASVSVLGDILGSAMQNTQNLLQMPYGCGEQNMVLFAPNIYVLDYLNETQQLTPEVKSKAIGYLNTGYQRQLNYKHYDGSYSTFGERYGRNQGNTWLTAFVLKTFAQARAYIFIDEAHITQALIWLSQRQKDNGCFRSSGSLLNNAIKGGVEDEVTLSAYITIALLEIPLTVTHPVVRNALFCLESAWKTAQEGDHGSHVYTKALLAYAFALAGNQDKRKEVLKSLNEEAVKKDNSVHWERPQKPKAPVGHFYEPQAPSAEVEMTSYVLLAYLTAQPAPTSEDLTSATNIVKWITKQQNAQGGFSSTQDTVVALHALSKYGAATFTRTGKAAQVTIQSSGTFSSKFQVDNNNRLLLQQVSLPELPGEYSMKVTGEGCVYLQTSLKYNILPEKEEFPFALGVQTLPQTCDEPKAHTSFQISLSVSYTGSRSASNMAIVDVKMVSGFIPLKPTVKMLERSNHVSRTEVSSNHVLIYLDKVSNQTLSLFFTVLQDVPVRDLKPAIVKVYDYYETDEFAIAEYNAPCSKDLGNA*

>A2M_TR_A21A

MGKNKLLHPSLVLLLLVLLPTDASVSGKPQYMVLVPSLLHTETTEKGCVLLSYLNETVTVSASLESVRGNRSLFTDLEAENDVLHCVAFAVPKSSSNEEVMFLTVQVKGPTQEFKKRTTVMVKNEDSLVFVQTDKSIYKPGQTVKFRVVSMDENFHPLNELIPLVYIQDPKGNRIAQWQSFQLEGGLKQFSFPLSSEPFQGSYKVVVQKKSGGRTEHPFTVEEFVLPKFEVQVTVPKIITILEEEMNVSVCGLYTYGKPVPGHVTVSICRKYSDASDCHGEDSQAFCEKFSGQLNSHGCFYQQVKTKVFQLKRKEYEMKLHTEAQIQEEGTVVELTGRQSSEITRTITKLSFVKVDSHFRQGIPFFGQVRLVDGKGVPIPNKVIFIRGNEANYYSNATTDEHGLVQFSINTTNVMGTSLTVRVNYKDRSPCYGYQWVSEEHEEAHHTAYLVFSPSKSFVHLEPMSHELPCGHTQTVQAHYILNGGTLLGLKKLSFYYLIMAKGGIVRTGTHGLLVKQEDMKGHFSISIPVKSDIAPVARLLIYAVLPTGDVIGDSAKYDVENCLANKVDLSFSPSQSLPASHAHLRVTAAPQSVCALRAVDQSVLLMKPDAELSASSVYNLLPEKDLTGFPGPLNDQDDEDCINRHNVYINGITYTPVSSTNEKDMYSFLEDMGLKAFTNSKIRKPKMCGGSGGSGGSGGSGGSGHMHAALTAGGSGGSGGSGGSGGSETVRKYFPETWIWDLVVVNSAGVAEVGVTVPDTITEWKAGAFCLSEDAGLGISSTASLRAFQPFFVELTMPYSVIRGEAFTLKATVLNYLPKCIRVSVQLEASPAFLAVPVEKEQAPHCICANGRQTVSWAVTPKSLGNVNFTVSAEALESQELCGTEVPSVPEHGRKDTVIKPLLVEPEGLEKETTFNSLLCPSGGEVSEELSLKLPPNVVEESARASVSVLGDILGSAMQNTQNLLQMPYGCGEQNMVLFAPNIYVLDYLNETQQLTPEVKSKAIGYLNTGYQRQLNYKHYDGSYSTFGERYGRNQGNTWLTAFVLKTFAQARAYIFIDEAHITQALIWLSQRQKDNGCFRSSGSLLNNAIKGGVEDEVTLSAYITIALLEIPLTVTHPVVRNALFCLESAWKTAQEGDHGSHVYTKALLAYAFALAGNQDKRKEVLKSLNEEAVKKDNSVHWERPQKPKAPVGHFYEPQAPSAEVEMTSYVLLAYLTAQPAPTSEDLTSATNIVKWITKQQNAQGGFSSTQDTVVALHALSKYGAATFTRTGKAAQVTIQSSGTFSSKFQVDNNNRLLLQQVSLPELPGEYSMKVTGEGCVYLQTSLKYNILPEKEEFPFALGVQTLPQTCDEPKAHTSFQISLSVSYTGSRSASNMAIVDVKMVSGFIPLKPTVKMLERSNHVSRTEVSSNHVLIYLDKVSNQTLSLFFTVLQDVPVRDLKPAIVKVYDYYETDEFAIAEYNAPCSKDLGNA*

>A2M_TR_B74

MGKNKLLHPSLVLLLLVLLPTDASVSGKPQYMVLVPSLLHTETTEKGCVLLSYLNETVTVSASLESVRGNRSLFTDLEAENDVLHCVAFAVPKSSSNEEVMFLTVQVKGPTQEFKKRTTVMVKNEDSLVFVQTDKSIYKPGQTVKFRVVSMDENFHPLNELIPLVYIQDPKGNRIAQWQSFQLEGGLKQFSFPLSSEPFQGSYKVVVQKKSGGRTEHPFTVEEFVLPKFEVQVTVPKIITILEEEMNVSVCGLYTYGKPVPGHVTVSICRKYSDASDCHGEDSQAFCEKFSGQLNSHGCFYQQVKTKVFQLKRKEYEMKLHTEAQIQEEGTVVELTGRQSSEITRTITKLSFVKVDSHFRQGIPFFGQVRLVDGKGVPIPNKVIFIRGNEANYYSNATTDEHGLVQFSINTTNVMGTSLTVRVNYKDRSPCYGYQWVSEEHEEAHHTAYLVFSPSKSFVHLEPMSHELPCGHTQTVQAHYILNGGTLLGLKKLSFYYLIMAKGGIVRTGTHGLLVKQEDMKGHFSISIPVKSDIAPVARLLIYAVLPTGDVIGDSAKYDVENCLANKVDLSFSPSQSLPASHAHLRVTAAPQSVCALRAVDQSVLLMKPDAELSASSVYNLLPEKDLTGFPGPLNDQDDEDCINRHNVYINGITYTPVSSTNEKDMYSFLEDMGLKAFTNSKIRKPKMCGGSGGSGGSGGSGGSGESLAYYTAGGSGGSGGSGGSGGSETVRKYFPETWIWDLVVVNSAGVAEVGVTVPDTITEWKAGAFCLSEDAGLGISSTASLRAFQPFFVELTMPYSVIRGEAFTLKATVLNYLPKCIRVSVQLEASPAFLAVPVEKEQAPHCICANGRQTVSWAVTPKSLGNVNFTVSAEALESQELCGTEVPSVPEHGRKDTVIKPLLVEPEGLEKETTFNSLLCPSGGEVSEELSLKLPPNVVEESARASVSVLGDILGSAMQNTQNLLQMPYGCGEQNMVLFAPNIYVLDYLNETQQLTPEVKSKAIGYLNTGYQRQLNYKHYDGSYSTFGERYGRNQGNTWLTAFVLKTFAQARAYIFIDEAHITQALIWLSQRQKDNGCFRSSGSLLNNAIKGGVEDEVTLSAYITIALLEIPLTVTHPVVRNALFCLESAWKTAQEGDHGSHVYTKALLAYAFALAGNQDKRKEVLKSLNEEAVKKDNSVHWERPQKPKAPVGHFYEPQAPSAEVEMTSYVLLAYLTAQPAPTSEDLTSATNIVKWITKQQNAQGGFSSTQDTVVALHALSKYGAATFTRTGKAAQVTIQSSGTFSSKFQVDNNNRLLLQQVSLPELPGEYSMKVTGEGCVYLQTSLKYNILPEKEEFPFALGVQTLPQTCDEPKAHTSFQISLSVSYTGSRSASNMAIVDVKMVSGFIPLKPTVKMLERSNHVSRTEVSSNHVLIYLDKVSNQTLSLFFTVLQDVPVRDLKPAIVKVYDYYETDEFAIAEYNAPCSKDLGNA*

>A2M_TR_C9

MGKNKLLHPSLVLLLLVLLPTDASVSGKPQYMVLVPSLLHTETTEKGCVLLSYLNETVTVSASLESVRGNRSLFTDLEAENDVLHCVAFAVPKSSSNEEVMFLTVQVKGPTQEFKKRTTVMVKNEDSLVFVQTDKSIYKPGQTVKFRVVSMDENFHPLNELIPLVYIQDPKGNRIAQWQSFQLEGGLKQFSFPLSSEPFQGSYKVVVQKKSGGRTEHPFTVEEFVLPKFEVQVTVPKIITILEEEMNVSVCGLYTYGKPVPGHVTVSICRKYSDASDCHGEDSQAFCEKFSGQLNSHGCFYQQVKTKVFQLKRKEYEMKLHTEAQIQEEGTVVELTGRQSSEITRTITKLSFVKVDSHFRQGIPFFGQVRLVDGKGVPIPNKVIFIRGNEANYYSNATTDEHGLVQFSINTTNVMGTSLTVRVNYKDRSPCYGYQWVSEEHEEAHHTAYLVFSPSKSFVHLEPMSHELPCGHTQTVQAHYILNGGTLLGLKKLSFYYLIMAKGGIVRTGTHGLLVKQEDMKGHFSISIPVKSDIAPVARLLIYAVLPTGDVIGDSAKYDVENCLANKVDLSFSPSQSLPASHAHLRVTAAPQSVCALRAVDQSVLLMKPDAELSASSVYNLLPEKDLTGFPGPLNDQDDEDCINRHNVYINGITYTPVSSTNEKDMYSFLEDMGLKAFTNSKIRKPKMCGGSGGSGGSGGSGGSGRSLSRLTAGGSGGSGGSGGSGGSETVRKYFPETWIWDLVVVNSAGVAEVGVTVPDTITEWKAGAFCLSEDAGLGISSTASLRAFQPFFVELTMPYSVIRGEAFTLKATVLNYLPKCIRVSVQLEASPAFLAVPVEKEQAPHCICANGRQTVSWAVTPKSLGNVNFTVSAEALESQELCGTEVPSVPEHGRKDTVIKPLLVEPEGLEKETTFNSLLCPSGGEVSEELSLKLPPNVVEESARASVSVLGDILGSAMQNTQNLLQMPYGCGEQNMVLFAPNIYVLDYLNETQQLTPEVKSKAIGYLNTGYQRQLNYKHYDGSYSTFGERYGRNQGNTWLTAFVLKTFAQARAYIFIDEAHITQALIWLSQRQKDNGCFRSSGSLLNNAIKGGVEDEVTLSAYITIALLEIPLTVTHPVVRNALFCLESAWKTAQEGDHGSHVYTKALLAYAFALAGNQDKRKEVLKSLNEEAVKKDNSVHWERPQKPKAPVGHFYEPQAPSAEVEMTSYVLLAYLTAQPAPTSEDLTSATNIVKWITKQQNAQGGFSSTQDTVVALHALSKYGAATFTRTGKAAQVTIQSSGTFSSKFQVDNNNRLLLQQVSLPELPGEYSMKVTGEGCVYLQTSLKYNILPEKEEFPFALGVQTLPQTCDEPKAHTSFQISLSVSYTGSRSASNMAIVDVKMVSGFIPLKPTVKMLERSNHVSRTEVSSNHVLIYLDKVSNQTLSLFFTVLQDVPVRDLKPAIVKVYDYYETDEFAIAEYNAPCSKDLGNA*

>A2M_TR_S1

MGKNKLLHPSLVLLLLVLLPTDASVSGKPQYMVLVPSLLHTETTEKGCVLLSYLNETVTVSASLESVRGNRSLFTDLEAENDVLHCVAFAVPKSSSNEEVMFLTVQVKGPTQEFKKRTTVMVKNEDSLVFVQTDKSIYKPGQTVKFRVVSMDENFHPLNELIPLVYIQDPKGNRIAQWQSFQLEGGLKQFSFPLSSEPFQGSYKVVVQKKSGGRTEHPFTVEEFVLPKFEVQVTVPKIITILEEEMNVSVCGLYTYGKPVPGHVTVSICRKYSDASDCHGEDSQAFCEKFSGQLNSHGCFYQQVKTKVFQLKRKEYEMKLHTEAQIQEEGTVVELTGRQSSEITRTITKLSFVKVDSHFRQGIPFFGQVRLVDGKGVPIPNKVIFIRGNEANYYSNATTDEHGLVQFSINTTNVMGTSLTVRVNYKDRSPCYGYQWVSEEHEEAHHTAYLVFSPSKSFVHLEPMSHELPCGHTQTVQAHYILNGGTLLGLKKLSFYYLIMAKGGIVRTGTHGLLVKQEDMKGHFSISIPVKSDIAPVARLLIYAVLPTGDVIGDSAKYDVENCLANKVDLSFSPSQSLPASHAHLRVTAAPQSVCALRAVDQSVLLMKPDAELSASSVYNLLPEKDLTGFPGPLNDQDDEDCINRHNVYINGITYTPVSSTNEKDMYSFLEDMGLKAFTNSKIRKPKMCGGSGGSGGSGGSGGGGSPQGIAGQGGSGGSGGSGGSGGSETVRKYFPETWIWDLVVVNSAGVAEVGVTVPDTITEWKAGAFCLSEDAGLGISSTASLRAFQPFFVELTMPYSVIRGEAFTLKATVLNYLPKCIRVSVQLEASPAFLAVPVEKEQAPHCICANGRQTVSWAVTPKSLGNVNFTVSAEALESQELCGTEVPSVPEHGRKDTVIKPLLVEPEGLEKETTFNSLLCPSGGEVSEELSLKLPPNVVEESARASVSVLGDILGSAMQNTQNLLQMPYGCGEQNMVLFAPNIYVLDYLNETQQLTPEVKSKAIGYLNTGYQRQLNYKHYDGSYSTFGERYGRNQGNTWLTAFVLKTFAQARAYIFIDEAHITQALIWLSQRQKDNGCFRSSGSLLNNAIKGGVEDEVTLSAYITIALLEIPLTVTHPVVRNALFCLESAWKTAQEGDHGSHVYTKALLAYAFALAGNQDKRKEVLKSLNEEAVKKDNSVHWERPQKPKAPVGHFYEPQAPSAEVEMTSYVLLAYLTAQPAPTSEDLTSATNIVKWITKQQNAQGGFSSTQDTVVALHALSKYGAATFTRTGKAAQVTIQSSGTFSSKFQVDNNNRLLLQQVSLPELPGEYSMKVTGEGCVYLQTSLKYNILPEKEEFPFALGVQTLPQTCDEPKAHTSFQISLSVSYTGSRSASNMAIVDVKMVSGFIPLKPTVKMLERSNHVSRTEVSSNHVLIYLDKVSNQTLSLFFTVLQDVPVRDLKPAIVKVYDYYETDEFAIAEYNAPCSKDLGNA*

>A2M_TR∆7_S1_I710

MGKNKLLHPSLVLLLLVLLPTDASVSGKPQYMVLVPSLLHTETTEKGCVLLSYLNETVTVSASLESVRGNRSLFTDLEAENDVLHCVAFAVPKSSSNEEVMFLTVQVKGPTQEFKKRTTVMVKNEDSLVFVQTDKSIYKPGQTVKFRVVSMDENFHPLNELIPLVYIQDPKGNRIAQWQSFQLEGGLKQFSFPLSSEPFQGSYKVVVQKKSGGRTEHPFTVEEFVLPKFEVQVTVPKIITILEEEMNVSVCGLYTYGKPVPGHVTVSICRKYSDASDCHGEDSQAFCEKFSGQLNSHGCFYQQVKTKVFQLKRKEYEMKLHTEAQIQEEGTVVELTGRQSSEITRTITKLSFVKVDSHFRQGIPFFGQVRLVDGKGVPIPNKVIFIRGNEANYYSNATTDEHGLVQFSINTTNVMGTSLTVRVNYKDRSPCYGYQWVSEEHEEAHHTAYLVFSPSKSFVHLEPMSHELPCGHTQTVQAHYILNGGTLLGLKKLSFYYLIMAKGGIVRTGTHGLLVKQEDMKGHFSISIPVKSDIAPVARLLIYAVLPTGDVIGDSAKYDVENCLANKVDLSFSPSQSLPASHAHLRVTAAPQSVCALRAVDQSVLLMKPDAELSASSVYNLLPEKDLTGFPGPLNDQDDEDCINRHNVYINGITYTPVSSTNEKDMYSFLEDMGLKAFTNSKIRKPKMCGGSGGSGGSGGSGGGGSPQGIAGQGGSGGSGGETVRKYFPETWIWDLVVVNSAGVAEVGVTVPDTITEWKAGAFCLSEDAGLGISSTASLRAFQPFFVELTMPYSVIRGEAFTLKATVLNYLPKCIRVSVQLEASPAFLAVPVEKEQAPHCICANGRQTVSWAVTPKSLGNVNFTVSAEALESQELCGTEVPSVPEHGRKDTVIKPLLVEPEGLEKETTFNSLLCPSGGEVSEELSLKLPPNVVEESARASVSVLGDILGSAMQNTQNLLQMPYGCGEQNMVLFAPNIYVLDYLNETQQLTPEVKSKAIGYLNTGYQRQLNYKHYDGSYSTFGERYGRNQGNTWLTAFVLKTFAQARAYIFIDEAHITQALIWLSQRQKDNGCFRSSGSLLNNAIKGGVEDEVTLSAYITIALLEIPLTVTHPVVRNALFCLESAWKTAQEGDHGSHVYTKALLAYAFALAGNQDKRKEVLKSLNEEAVKKDNSVHWERPQKPKAPVGHFYEPQAPSAEVEMTSYVLLAYLTAQPAPTSEDLTSATNIVKWITKQQNAQGGFSSTQDTVVALHALSKYGAATFTRTGKAAQVTIQSSGTFSSKFQVDNNNRLLLQQVSLPELPGEYSMKVTGEGCVYLQTSLKYNILPEKEEFPFALGVQTLPQTCDEPKAHTSFQISLSVSYTGSRSASNMAIVDVKMVSGFIPLKPTVKMLERSNHVSRTEVSSNHVLIYLDKVSNQTLSLFFTVLQDVPVRDLKPAIVKVYDYYETDEFAIAEYNAPCSKDLGNA*

>A2M_TR_S1_QRT4

MGKNKLLHPSLVLLLLVLLPTDASVSGKPQYMVLVPSLLHTETTEKGCVLLSYLNETVTVSASLESVRGNRSLFTDLEAENDVLHCVAFAVPKSSSNEEVMFLTVQVKGPTQEFKKRTTVMVKNEDSLVFVQTDKSIYKPGQTVKFRVVSMDENFHPLNELIPLVYIQDPKGNRIAQWQSFQLEGGLKQFSFPLSSEPFQGSYKVVVQKKSGGRTEHPFTVEEFVLPKFEVQVTVPKIITILEEEMNVSVCGLYTYGKPVPGHVTVSICRKYSDASDCHGEDSQAFCEKFSGQLNSHGCFYQQVKTKVFQLKRKEYEMKLHTEAQIQEEGTVVELTGRQSSEITRTITKLSFVKVDSHFRQGIPFFGQVRLVDGKGVPIPNKVIFIRGNEANYYSNATTDEHGLVQFSINTTNVMGTSLTVRVNYKDRSPCYGYQWVSEEHEEAHHTAYLVFSPSKSFVHLEPMSHELPCGHTQTVQAHYILNGGTLLGLKKLSFYYLIMAKGGIVRTGTHGLLVKQEDMKGHFSISIPVKSDIAPVARLLIYAVLPTGDVIGDSAKYDVENCLANKVDLSFSPSQSLPASHAHLRVTAAPQSVCALRAVDQSVLLMKPDAELSASSVYNLLPEKDLTGFPGPLNDQDDEDCINRHNVYINGITYTPVSSTNEKDMYSFLEDMGLKAFTNSKIRKPKMCGGSGGSGGSGGSGGGGSPQGIAGQGGSGGLVHVEEPHTETVRKYFPETWIWDLVVVNSAGVAEVGVTVPDTITEWKAGAFCLSEDAGLGISSTASLRAFQPFFVELTMPYSVIRGEAFTLKATVLNYLPKCIRVSVQLEASPAFLAVPVEKEQAPHCICANGRQTVSWAVTPKSLGNVNFTVSAEALESQELCGTEVPSVPEHGRKDTVIKPLLVEPEGLEKETTFNSLLCPSGGEVSEELSLKLPPNVVEESARASVSVLGDILGSAMQNTQNLLQMPYGCGEQNMVLFAPNIYVLDYLNETQQLTPEVKSKAIGYLNTGYQRQLNYKHYDGSYSTFGERYGRNQGNTWLTAFVLKTFAQARAYIFIDEAHITQALIWLSQRQKDNGCFRSSGSLLNNAIKGGVEDEVTLSAYITIALLEIPLTVTHPVVRNALFCLESAWKTAQEGDHGSHVYTKALLAYAFALAGNQDKRKEVLKSLNEEAVKKDNSVHWERPQKPKAPVGHFYEPQAPSAEVEMTSYVLLAYLTAQPAPTSEDLTSATNIVKWITKQQNAQGGFSSTQDTVVALHALSKYGAATFTRTGKAAQVTIQSSGTFSSKFQVDNNNRLLLQQVSLPELPGEYSMKVTGEGCVYLQTSLKYNILPEKEEFPFALGVQTLPQTCDEPKAHTSFQISLSVSYTGSRSASNMAIVDVKMVSGFIPLKPTVKMLERSNHVSRTEVSSNHVLIYLDKVSNQTLSLFFTVLQDVPVRDLKPAIVKVYDYYETDEFAIAEYNAPCSKDLGNA*

>A2M_TR∆7_S1_I703

MGKNKLLHPSLVLLLLVLLPTDASVSGKPQYMVLVPSLLHTETTEKGCVLLSYLNETVTVSASLESVRGNRSLFTDLEAENDVLHCVAFAVPKSSSNEEVMFLTVQVKGPTQEFKKRTTVMVKNEDSLVFVQTDKSIYKPGQTVKFRVVSMDENFHPLNELIPLVYIQDPKGNRIAQWQSFQLEGGLKQFSFPLSSEPFQGSYKVVVQKKSGGRTEHPFTVEEFVLPKFEVQVTVPKIITILEEEMNVSVCGLYTYGKPVPGHVTVSICRKYSDASDCHGEDSQAFCEKFSGQLNSHGCFYQQVKTKVFQLKRKEYEMKLHTEAQIQEEGTVVELTGRQSSEITRTITKLSFVKVDSHFRQGIPFFGQVRLVDGKGVPIPNKVIFIRGNEANYYSNATTDEHGLVQFSINTTNVMGTSLTVRVNYKDRSPCYGYQWVSEEHEEAHHTAYLVFSPSKSFVHLEPMSHELPCGHTQTVQAHYILNGGTLLGLKKLSFYYLIMAKGGIVRTGTHGLLVKQEDMKGHFSISIPVKSDIAPVARLLIYAVLPTGDVIGDSAKYDVENCLANKVDLSFSPSQSLPASHAHLRVTAAPQSVCALRAVDQSVLLMKPDAELSASSVYNLLPEKDLTGFPGPLNDQDDEDCINRHNVYINGITYTPVSSTNEKDMYSFLEDMGLKAFTNSKIRKPKMCGGSGGSGGSGPQGIAGQSGGSGGSGGSGGSGGETVRKYFPETWIWDLVVVNSAGVAEVGVTVPDTITEWKAGAFCLSEDAGLGISSTASLRAFQPFFVELTMPYSVIRGEAFTLKATVLNYLPKCIRVSVQLEASPAFLAVPVEKEQAPHCICANGRQTVSWAVTPKSLGNVNFTVSAEALESQELCGTEVPSVPEHGRKDTVIKPLLVEPEGLEKETTFNSLLCPSGGEVSEELSLKLPPNVVEESARASVSVLGDILGSAMQNTQNLLQMPYGCGEQNMVLFAPNIYVLDYLNETQQLTPEVKSKAIGYLNTGYQRQLNYKHYDGSYSTFGERYGRNQGNTWLTAFVLKTFAQARAYIFIDEAHITQALIWLSQRQKDNGCFRSSGSLLNNAIKGGVEDEVTLSAYITIALLEIPLTVTHPVVRNALFCLESAWKTAQEGDHGSHVYTKALLAYAFALAGNQDKRKEVLKSLNEEAVKKDNSVHWERPQKPKAPVGHFYEPQAPSAEVEMTSYVLLAYLTAQPAPTSEDLTSATNIVKWITKQQNAQGGFSSTQDTVVALHALSKYGAATFTRTGKAAQVTIQSSGTFSSKFQVDNNNRLLLQQVSLPELPGEYSMKVTGEGCVYLQTSLKYNILPEKEEFPFALGVQTLPQTCDEPKAHTSFQISLSVSYTGSRSASNMAIVDVKMVSGFIPLKPTVKMLERSNHVSRTEVSSNHVLIYLDKVSNQTLSLFFTVLQDVPVRDLKPAIVKVYDYYETDEFAIAEYNAPCSKDLGNA*
